# Global, regional, and national burden of Guillain–Barré syndrome and its underlying causes from 1990 to 2019

**DOI:** 10.1186/s12974-021-02319-4

**Published:** 2021-11-11

**Authors:** Nicola Luigi Bragazzi, Ali-Asghar Kolahi, Seyed Aria Nejadghaderi, Piergiorgio Lochner, Francesco Brigo, Andrea Naldi, Paola Lanteri, Sergio Garbarino, Mark J. M. Sullman, Haijiang Dai, Jianhong Wu, Jude Dzevela Kong, Haitham Jahrami, Mohammad-Reza Sohrabi, Saeid Safiri

**Affiliations:** 1 Centre for Disease Modelling, York University, Toronto, ON Canada; 2 Social Determinants of Health Research Center, Shahid Beheshti University of Medical Sciences, Tehran, Iran; 3 Systematic Review and Meta-Analysis Expert Group (SRMEG), Universal Scientific Education and Research Network (USERN), Tehran, Iran; 4 Department of Neurology, Saarland University Medical Center, Homburg, Germany; 5 Department of Neurology, Franz Tappeiner Hospital, Merano, Italy; 6 Department of Neuroscience “Rita Levi Montalcini”, University of Turin, Turin, Italy; 7 Neurophysiology Unit, Fondazione IRCCS Istituto Neurologico Carlo Besta, Milan, Italy; 8 Department of Neuroscience, Rehabilitation, Ophthalmology, Genetics and Maternal/Child Sciences (DINOGMI), Polyclinic Hospital San Martino IRCCS, University of Genoa, Genoa, Italy; 9 Department of Social Sciences, University of Nicosia, Nicosia, Cyprus; 10 Department of Life and Health Sciences, University of Nicosia, Nicosia, Cyprus; 11 College of Medicine and Medical Sciences, Arabian Gulf University, Manama, Bahrain; 12 Neurosciences Research Center, Aging Research Institute, Tabriz University of Medical Sciences, Tabriz, Iran; 13 Department of Community Medicine, Faculty of Medicine, Tabriz University of Medical Sciences, Tabriz, Iran

**Keywords:** Global burden of disease, Guillain–Barré syndrome, Prevalence, Years lived with disability, Cause

## Abstract

**Background:**

This article presents the first detailed analysis of the prevalence and disability burden of Guillain–Barré syndrome (GBS) from 1990 to 2019 by cause, age, sex, and Socio-demographic Index (SDI) in 204 countries and territories.

**Methods:**

Data from the Global Burden of Diseases Study (GBD) 2019 were used. GBD 2019 modelled the prevalence of GBS using hospital and claims data. Years lived with disability (YLDs) were estimated as the product of the GBS prevalence and the disability weight. This article also reported proportions in the age-standardised prevalence rate that were due to six underlying causes of GBS.

**Results:**

In 2019, there were 150,095 [95% uncertainty intervals (UI) 119,924 to 188,309] total cases of GBS worldwide, which resulted in 44,407 (95% UI 28,016 to 64,777) YLDs. Globally, there was a 6.4% (95% UI 3.6 to 9.5) increase in the age-standardised prevalence of GBS per 100,000 population between 1990 and 2019. High-income Asia Pacific [1.9 (95% UI: 1.5 to 2.4)] and East Asia [0.8 (95% UI: 0.6 to 1.0)] had the highest and lowest age-standardised prevalence rates (per 100,000), respectively, in 2019. Nationally, Japan [6.4 (95% UI: 5.3 to 7.7)] and China [0.8 (95% UI: 0.6 to 1.0)] had the highest and lowest age-standardised prevalence rates (per 100,000). The age-standardised burden of GBS increased with increasing age and was higher in males in all age groups. Furthermore, the age-standardised prevalence of GBS (per 100,000) had a positive association with the level of development, as measured by SDI, although this association was not strong. Upper respiratory infections and unknown causes accounted for the highest proportions of underlying causes.

**Conclusions:**

Globally, the prevalence of GBS continues to increase. Geographical differences and strategies aimed at preventing infectious diseases should be considered in future health policy planning and decision-making processes. This study had several limitations, such as using the same disability weight for all causes and a reliance on hospital- and self-reported data, which should be addressed in future research.

**Supplementary Information:**

The online version contains supplementary material available at 10.1186/s12974-021-02319-4.

## Introduction

Guillain–Barré syndrome (GBS) is an acute immune-mediated polyneuropathy. It represents an aberrant autoimmune response to a preceding infection or other immune stimulation, which leads the immune system to attack the myelin sheaths or axons of the peripheral nerves and their spinal roots, due to molecular mimicry [1, 2]. GBS occurs worldwide with an overall incidence rate of 1–2 cases per 100,000 people per year, affecting all age groups, but is slightly more common in males than in females [3–5]. Mortality, or severe disability due to GBS, occurs in around 20% of patients [6].

Clinically, GBS manifests itself with progressive muscle weakness associated with decreased or absent deep tendon reflexes, as well as mild to severe sensory signs and symptoms [1]. Symptoms usually start in the lower limbs and gradually ascend to involve the arms and facial muscles [1]. Dysfunction in the autonomic system occurs in approximately 70% of patients and can lead to death [7]. GBS follows a monophasic course, usually progressing over a period of about 2 weeks, with symptoms reaching the nadir around 4 weeks after onset. Most patients require hospitalization and some of them ventilator assistance in intensive care units [8]. Overall, more than 80% have a complete or nearly complete recovery; the prognosis is worse in elderly patients and in cases with axonal involvement [2]. However, some patients have a protracted recovery, resulting in disability which can be permanent or lasting several years [1]. Respiratory or gastrointestinal infection, infection with the Zika virus, and autoimmunity are some of the suggested etiologies for the development of GBS [6].

To develop and implement specific strategies aimed at improving the health outcomes for people with GBS, it is important to systematically evaluate the burden of this condition. However, to the best of our knowledge, no prior research has provided estimates for the burden and trends associated with GBS at the global level. The primary aim of this report was to present the first detailed analysis of the prevalence and disability burden of GBS by cause, age, sex, and Socio-demographic Index (SDI) in 204 countries and territories from 1990 to 2019, using data from the Global Burden of Diseases Study (GBD) 2019.

## Methods

### Overview

This study is part of the GBD 2019 project, which was conducted by the Institute for Health Metrics and Evaluation (IHME) to provide a systematic investigation of the burden of 369 diseases and injuries and 87 behavioural, environmental, occupational, and metabolic risk factors [9–11]. GBD 2019 included data for seven super-regions, 21 regions, and 204 countries and territories from 1990 to 2019. Full details of the methods used in GBD 2019 are described in existing GBD literature [9–11]. As GBD 2019 used de-identified aggregated data, a waiver of informed consent was reviewed and approved by the University of Washington, Seattle, Washington, United States of America, Institutional Review Board. All data on the burden of GBS, for all countries and regions and from the period 1990 to 2019, are publicly available on the IHME website [Available from: http://ghdx.healthdata.org/gbd-results-tool].

### Data sources

The International Classification of Disease (ICD) codes of G61.0 (GBS) and 357.0 (Acute infective polyneuritis) were considered by IHME to be GBS. In this study, inpatient hospital data were extracted using the ICD codes listed above. Only primary diagnoses were considered, with the reasoning being that GBS should appear as a primary diagnosis and IHME did not wish to include follow-up visits that may be listed as secondary or tertiary codes. In addition, two additional years of claims data from the United States of America (USA) (2015, 2016) and 3 years of claims data from Poland (2015, 2016, 2017), for the first time, were also included in GBD 2019 [9]. More detailed information on the data sources used for the estimations of GBS can be found on the GBD 2019 Data Input Sources Tool website [http://ghdx.healthdata.org/gbd-2019/data-input-sources].

### Prevalence and underlying cause estimation

The prevalence of GBS for each location, year, age, and sex were estimated using DisMod-MR 2.1, a Bayesian meta-regression tool developed for GBD analyses [12]. Unlike the last GBD iteration, the hospital data were not adjusted in this study, as they were quite similar to the data from the literature. To divide the overall prevalence of GBS according to the underlying cause, a systematic review of the literature was conducted to identify studies on the proportion of GBS cases attributable to any described aetiological cause, including influenza, upper respiratory infections, diarrheal diseases, and other infectious diseases. Based on these studies, an aetiological proportion model was developed using DisMod-MR 2.1 and used to divide the prevalent cases of GBS by cause. A random effects meta-analysis was used to pool these proportions. The proportions for influenza, upper respiratory infections, diarrheal diseases, and other infectious diseases were squeezed to add to the proportion for all identified underlying infectious diseases [12]. Finally, the remaining proportion with any underlying infectious disease were assigned to the “idiopathic GBS” category, which is classified under unknown causes [12].

### YLDs estimation

To calculate the YLDs for GBS, the prevalence of GBS (in number of cases) was multiplied by their disability weight, which quantifies the magnitude of health loss associated with GBS. The YLD rate is defined as the number of YLDs expressed per 100,000 population. Disability weights are measured on a scale from 0 to 1, where 0 represents full health and 1 is equivalent to death. More information about the process of disability weight estimation has been described in detail elsewhere [9, 10]. In GBD 2019, GBS attributed causes (i.e., lower respiratory infections, upper respiratory infections, diarrheal diseases and other infectious diseases) were all assigned the same disability weight, which was 0.296 (0.198–0.414) (Additional file 1: Table S1) [9].

### Socio-demographic Index (SDI)

We used SDI to explore the relationship that the development level of a region or country has with GBS prevalence and YLDs [9]. Smoothing splines models were used to examine the association between the burden of GBS, computed in terms of YLDs, and SDI for 21 regions and 204 countries and territories [13]. The SDI, a composite indicator that quantifies the development level, ranges from 0 (the worst) to 1 (the best). It is calculated based on the average educational attainment in the population aged 15 years or older, total fertility rate under 25 years, and lag-distributed income per capita. The cutoff values used to determine SDI quintiles were computed using estimates from countries with populations over 1 million. The 204 countries and territories were divided into five groups, according to SDI quintile: low SDI, low-middle SDI, middle SDI, high-middle SDI, and high SDI [9].

### Uncertainty analysis

IHME propagated uncertainty through all calculations by sampling 1000 draws at each step of the calculations [9]. Final estimates were determined using the mean estimate across 1000 draws, and the 95% uncertainty intervals (UIs) were defined as the 25th and 975th values of the 1000 ordered draws. For all estimates, a 95% UI excluding zero was considered to be statistically significant.

## Results

### Global level

In 2019, there were 150,095 (95% UI: 119,924 to 188,309) cases of GBS globally, with an age-standardised point prevalence of 1.9 per 100,000 population (95% UI: 1.5 to 2.4), which represents a 6.4% increase since 1990 (95% UI: 3.6 to 9.5). GBS accounted for 44,407 (95% UI: 28,016 to 64,777) YLDs in 2019, with an age-standardised rate of 0.6 (95% UI: 0.4 to 0.8), which has increased 6.5% since 1990 (95% UI: 3.6 to 9.5) (Table 1).

**Table 1:** Prevalent cases and years lived with disability (YLDs) for Guillain–Barre syndrome in 2019 and the percentage change in the age-standardised rates (ASRs) per 100,000, by GBD region, from 1990 to 2019 (Generated from data available from http://ghdx.healthdata.org/gbd-results-tool)

	Prevalence (95% UI)			YLD (95% UI)		
	No (95% UI)	ASRs per 100,000 (95% UI)	Percentage change in ASRs between 1990 and 2019	No (95% UI)	ASRs per 100,000 (95% UI)	Percentage change in ASRs between 1990 and 2019
Global	150,095 (119,924, 188,309)	1.9 (1.5, 2.4)	6.4 (3.6, 9.5)	44,407 (28,016, 64,777)	0.6 (0.4, 0.8)	6.5 (3.6, 9.5)
High-income Asia Pacific	11,708 (9631, 14,284)	6.4 (5.2, 7.7)	9.3 (4, 14.8)	3465 (2187, 5008)	1.9 (1.2, 2.8)	9.3 (4, 14.8)
High-income North America	20,834 (16,851, 25,728)	4.2 (3.5, 5.1)	41.2 (28.8, 56.1)	6166 (3847, 8752)	1.3 (0.8, 1.8)	41.3 (28.8, 56.1)
Western Europe	11,188 (8605, 14,514)	1.9 (1.5, 2.4)	17 (14.1, 20.6)	3311 (1985, 4876)	0.6 (0.4, 0.8)	17 (14.1, 20.6)
Australasia	560 (431, 726)	1.6 (1.3, 2.1)	13.8 (5, 24.1)	166 (100, 245)	0.5 (0.3, 0.7)	13.8 (5, 24.1)
Andean Latin America	1373 (1096, 1717)	2.3 (1.8, 2.8)	− 6.8 (− 8.6, − 4.7)	406 (255, 592)	0.7 (0.4, 1)	− 6.8 (− 8.6, − 4.7)
Tropical Latin America	3205 (2530, 4006)	1.4 (1.1, 1.7)	− 40.3 (− 49.9, − 31.3)	948 (581, 1399)	0.4 (0.2, 0.6)	− 40.3 (− 49.9, − 31.3)
Central Latin America	9501 (7587, 11,892)	3.9 (3.1, 4.9)	2.7 (− 0.9, 7.1)	2811 (1742, 4128)	1.2 (0.7, 1.7)	2.7 (− 0.9, 7.1)
Southern Latin America	1764 (1371, 2270)	2.5 (1.9, 3.2)	0.1 (0.1, 0.2)	522 (317, 767)	0.7 (0.4, 1.1)	0.1 (0.1, 0.2)
Caribbean	980 (771, 1248)	2 (1.6, 2.6)	0 (− 0.1, 0.1)	290 (180, 427)	0.6 (0.4, 0.9)	0 (− 0.1, 0.1)
Central Europe	1858 (1424, 2429)	1.4 (1.1, 1.8)	− 1.9 (− 5.6, 2.7)	550 (331, 807)	0.4 (0.3, 0.6)	− 1.9 (− 5.6, 2.7)
Eastern Europe	4323 (3349, 5635)	1.9 (1.5, 2.4)	0.4 (0.2, 0.6)	1279 (791, 1896)	0.6 (0.3, 0.8)	0.4 (0.2, 0.6)
Central Asia	1695 (1314, 2135)	1.9 (1.5, 2.4)	0 (− 0.7, 0.6)	501 (313, 751)	0.6 (0.3, 0.8)	0 (− 0.7, 0.6)
North Africa and Middle East	10,386 (8094, 13,324)	1.8 (1.4, 2.3)	0.3 (− 0.9, 1.5)	3073 (1903, 4538)	0.5 (0.3, 0.8)	0.3 (− 0.9, 1.5)
South Asia	30,312 (23,426, 38,597)	1.8 (1.4, 2.2)	1.4 (− 2.9, 5.6)	8967 (5591, 13,346)	0.5 (0.3, 0.8)	1.4 (− 2.9, 5.6)
Southeast Asia	7087 (5401, 9186)	1.1 (0.8, 1.4)	0.3 (0.1, 0.6)	2096 (1299, 3172)	0.3 (0.2, 0.5)	0.3 (0.1, 0.6)
East Asia	11,886 (8933, 15,912)	0.8 (0.6, 1)	11.4 (4.7, 18.7)	3515 (2120, 5320)	0.2 (0.1, 0.4)	11.4 (4.7, 18.7)
Oceania	127 (94, 167)	1 (0.8, 1.4)	0 (− 0.1, 0)	38 (23, 58)	0.3 (0.2, 0.5)	0 (− 0.1, 0)
Western Sub-Saharan Africa	10,306 (7889, 13,113)	2.5 (2, 3.2)	− 0.4 (− 0.9, 0)	3049 (1895, 4575)	0.7 (0.5, 1.1)	− 0.4 (− 0.9, 0)
Eastern Sub-Saharan Africa	5580 (4169, 7187)	1.5 (1.2, 2)	1.7 (0.5, 2.9)	1651 (1016, 2509)	0.5 (0.3, 0.7)	1.7 (0.5, 2.9)
Central Sub-Saharan Africa	2836 (2158, 3624)	2.4 (1.9, 3)	− 0.2 (− 0.5, 0)	839 (522, 1257)	0.7 (0.4, 1.1)	− 0.2 (− 0.5, 0)
Southern Sub-Saharan Africa	2585 (2032, 3229)	3.4 (2.7, 4.2)	− 0.1 (− 0.3, 0)	765 (478, 1144)	1 (0.6, 1.5)	− 0.1 (− 0.3, 0)

### Regional level

In 2019, the age-standardised point prevalence of GBS (per 100,000 population) was highest in High-income Asia Pacific [6.4 (95% UI: 5.2 to 7.7)], High-income North America [4.2 (95% UI: 3.5 to 5.1)] and Central Latin America [3.9 (95% UI: 3.1 to 4.9)]. East Asia [0.8 (95% UI: 0.6 to 1.0)], Oceania [1.0 (95% UI: 0.8 to 1.4)] and Southeast Asia [1.1 (95% UI: 0.8 to 1.4)] had the lowest age-standardised rates (Table 1).

High-income Asia Pacific [1.9 (95% UI: 1.2 to 2.8)], High-income North America [1.3 (95% UI: 0.8 to 1.8)] and Central Latin America [1.2 (95% UI: 0.7 to 1.7)] had the highest age-standardised YLD rates from GBS. The rates were lowest for East Asia [0.2 (95% UI: 0.1 to 0.4)], Oceania [0.3 (95% UI: 0.2 to 0.5)] and Southeast Asia [0.3 (95% UI: 0.2 to 0.5)] (Table 1). The age-standardised point prevalence and YLD rates of GBS, for all GBD regions in 2019, are presented in Additional file 4: Figures S1 and Additional file 5: S2, respectively.

Most regions showed an increase in the age-standardised point prevalence of GBS, from 1990 to 2019, except Tropical Latin America [− 40.3% (95% UI: − 49.9 to − 31.3)] and Andean Latin America [− 6.8% (95% UI: − 8.6 to − 4.7)] (Table 1). In the same period, most regions showed an increase in the age-standardised YLD rates of GBS from, except Tropical Latin America [− 40.3% (95% UI: − 49.9 to − 31.3)] and Andean Latin America [− 6.8% (95% UI: − 8.6 to − 4.7)] (Table 1). The percentage change, from 1990 to 2019, in the age-standardised point prevalence and YLD rates for GBS are presented in Additional file 6: Fig. S3 and Additional file 7: Fig. S4, respectively.

The global number of cases of GBS increased from 90,249 (95% UI: 70,747 to 114,487) in 1990 to 150,095 (95% UI: 119,924 to 188,309) in 2019. South Asia, High-income North America and East Asia experienced the largest number of cases in 2019 (Additional file 2: Table S2). The global number of YLDs due to GBS increased from 26,696 (95% UI: 16,714 to 39,628) in 1990 to 44,407 (95% UI: 28,016 to 64,777) in 2019, with South Asia, High-income North America and East Asia having the highest numbers of YLDs, due to GBS, in 2019 (Additional file 3: Table S3).

### National level

**Fig. 1:**
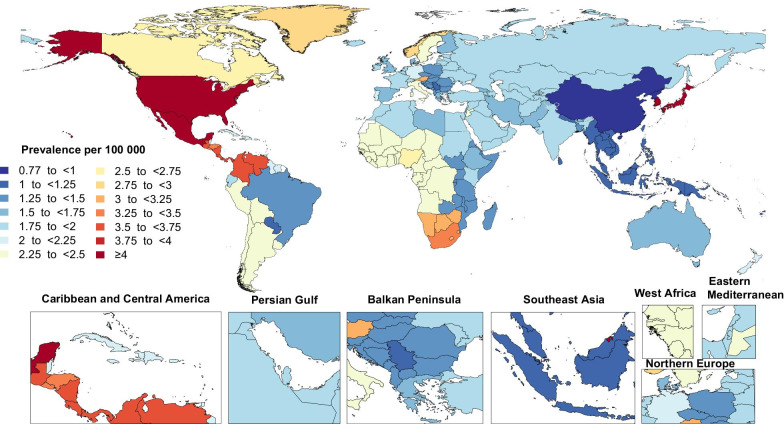
Age-standardised point prevalence of Guillain–Barre syndrome per 100,000 population in 2019, by country. (Generated from data available from http://ghdx.healthdata.org/gbd-results-tool)

In 2019, the national age-standardised point prevalence of GBS ranged from 0.8 to 6.4 cases per 100,000 population. Japan [6.4 (95% UI: 5.3 to 7.7)], Brunei Darussalam [6.3 (95% UI: 5.0 to 7.8)] and Singapore [6.3 (95% UI: 5.0 to 7.8)] had the highest age-standardised point prevalences (per 100,000) of GBS in 2019. In contrast, China [0.8 (95% UI: 0.6 to 1.0)], the Democratic People's Republic of Korea [0.9 (95% UI: 0.6 to 1.1)], and Kiribati [1.0 (95% UI: 0.8 to 1.3)] had the lowest point prevalences (per 100,000) (Fig. 1 and Additional file 2: Table S2).

**Fig. 2:**
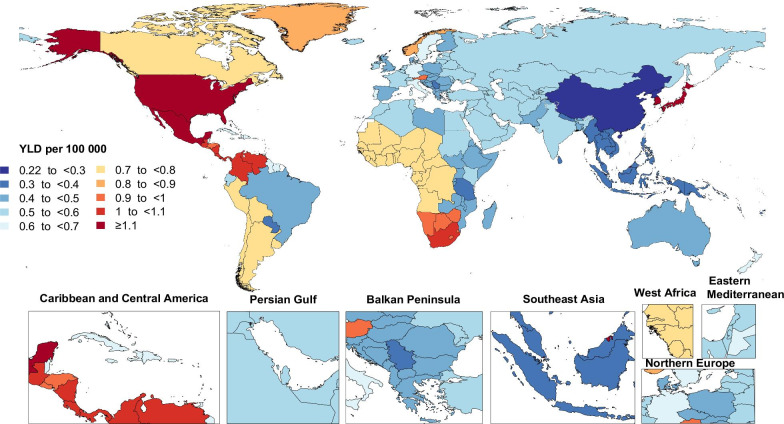
Age-standardised years lived with disability (YLDs) rate of Guillain–Barre syndrome per 100,000 population in 2019, by country. (Generated from data available from http://ghdx.healthdata.org/gbd-results-tool)

The national age-standardised YLD rates of GBS varied in 2019 from 0.2 to 1.9 cases per 100,000 population. The highest YLD rates (per 100,000) were observed in Japan [1.9 (95% UI: 1.2 to 2.8)], Brunei Darussalam [1.9 (95% UI: 1.2 to 2.7)] and Singapore [1.9 (95% UI: 1.2 to 2.7)], while the lowest YLD rates (per 100,000) were found in China [0.2 (95% UI: 0.1 to 0.4)], the Democratic People’s Republic of Korea [0.3 (95% UI: 0.2 to 0.4)], and Kiribati [0.3 (95% UI: 0.2 to 0.5] (Fig. 2 and Additional file 3: Table S3).

The percentage change in the age-standardised point prevalence (per 100,000), from 1990 to 2019, differed substantially between countries, with the United Kingdom [104.7% (95% UI: 90.1 to 124.4)], Sweden [102.6% (95% UI: 85.8 to 122.4)] and Austria [53.5% (95% UI: 32.4 to 83.0)] showing the largest increases during the measurement period. In contrast, Brazil [− 40.7% (95% UI: − 50.4 to − 31.6)], Ecuador [− 26.1% (95% UI: − 32.0 to − 19.0)] and Nepal [− 15.7% (95% UI: − 22.5 to − 7.4)] showed the largest decreases in the age-standardised point prevalence (per 100,000) (Additional file 2: Table S2).

The United Kingdom [104.7% (95% UI: 90.1 to 124.4)], Sweden [102.6% (95% UI: 85.8 to 122.4)] and Austria [53.5% (95% UI: 32.4 to 83.0)] showed the largest increases in the age-standardised YLD rates (per 100,000) of GBS over the measurement period (Additional file 3: Table S3). Conversely, Brazil [− 40.7% (95% UI: − 50.4 to − 31.6)], Ecuador [− 26.1% (95% UI: − 32.0 to − 19.0)] and Nepal [− 15.8% (95% UI: − 22.5 to − 7.4)] showed the largest decreases in YLDs (per 100,000) over the same period (Additional file 3: Table S3).

### Age and sex patterns

**Fig. 3:**
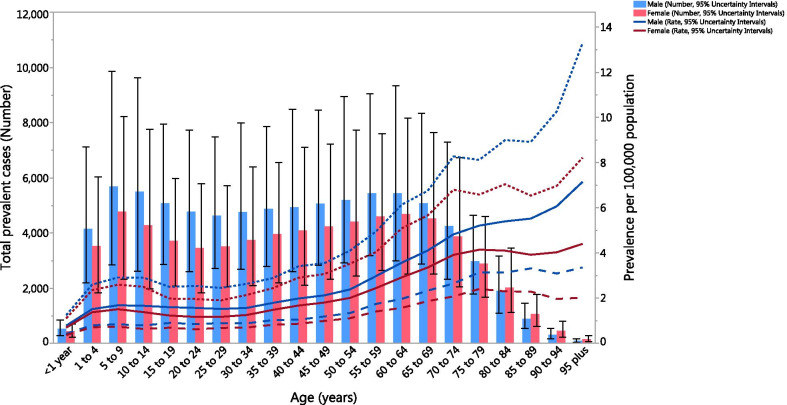
Global total number of cases and the prevalence of Guillain–Barre syndrome per 100,000 population, by age and sex in 2019. The error bars represent 95% uncertainty intervals for total number of cases. Dotted and dashed lines indicate 95% uncertainty intervals for prevalence (per 100,000). (Generated from data available from http://ghdx.healthdata.org/gbd-results-tool)

In 2019, the global point prevalence of GBS (per 100,000) broadly showed an increase with advancing age. More specifically, the total number of cases was highest in the 5–9 year age group, but decreased from there to the 25–29 age group, then increased up to the 60–64 age group, before decreasing again to the oldest age group. Furthermore, the global point prevalence of GBS (per 100,000) was higher in males in all age groups, while the total number of cases was higher in males up to the 75–79 age group, after which the pattern was reversed (Fig. 3).

In 2019, the global YLD rate of GBS (per 100,000) was highest in those aged 95 + years and was higher among males. However, the total number of YLDs was highest in the 5–9 age group for both males and females (Additional file 8: Figure S5).

### Association with the Socio-demographic Index (SDI)

**Fig. 4:**
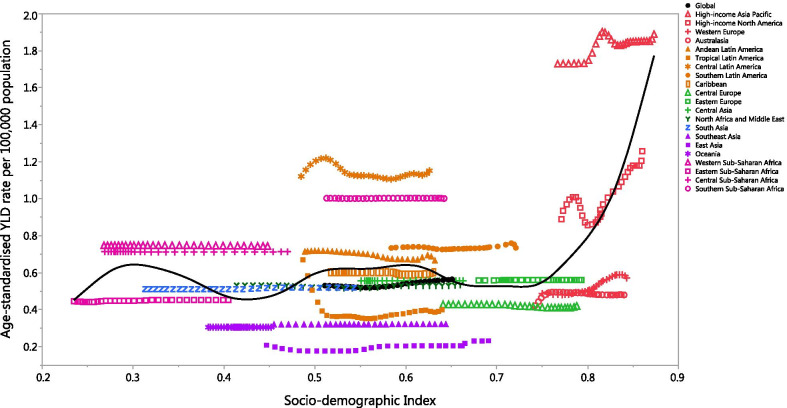
Age-standardised years lived with disability (YLDs) rates of Guillain–Barre syndrome for 204 countries and territories, by Socio-demographic Index (SDI) in 2019; Expected values based on the Socio-demographic Index and disease rates in all locations are shown as the black line. Each point shows the observed age-standardised YLD rate for each country in 2019. (Generated from data available from http://ghdx.healthdata.org/gbd-results-tool)

At the regional level there was a positive association between SDI and the age-standardised YLD rate of GBS, suggesting that the burden of GBS was higher in regions with higher socio-economic development. High-income Asia Pacific, Central Latin America, Southern Sub-Saharan Africa, Western Sub-Saharan Africa, Central Sub-Saharan Africa, Southern Latin America and Andean Latin America had higher than expected YLD rates, from 1990 to 2019, based upon their level of socio-demographic development (as measured by the SDI). In contrast, Western Europe, Central Europe, Australasia, Southeast Asia, East Asia and Oceania had lower than expected burdens from 1990 to 2019 (Fig. 4).

At the country-level, in 2019 the burden of GBS increased with increasing socio-economic development, up to an SDI of around 0.3, but then decreased slightly up to an SDI of about 0.7, then increased up to an SDI of 0.9, before decreasing again (Additional file 9: Figure S6). Countries and territories, such as Japan, Brunei Darussalam, Singapore, the Republic of Korea, the USA and Mexico had much higher than expected burdens, whereas countries and territories such as China, Fiji, Taiwan and Guam had much lower than expected burdens (Additional file 9: Fig. S6).

The global point prevalence of GBS was stable from 1990 to 2000, and from 2000 it began to gradually increase. Up to about 2010, the low SDI quintile and high SDI quintile both had point prevalence rates which were higher than the global level, while middle, low–middle and high–middle SDI quintiles had rates which were lower than the global level. Furthermore, the age-standardised point prevalence of GBS was stable in all SDI quintiles over 1990–2019 except the high-SDI quintile, which had an increase in its point prevalence rate from 2005 (Additional file 10: Fig. S7). The age-standardised YLD rates had a similar pattern to the point prevalence rates for the different SDI quintiles (Additional file 11: Fig. S8).

### Underlying causes

**Fig. 5:**
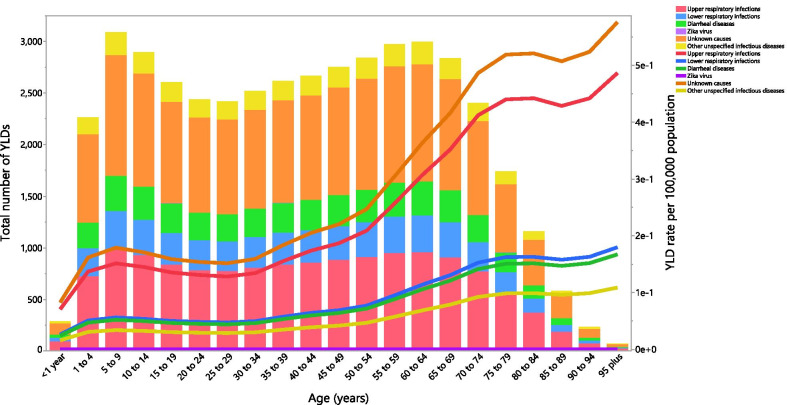
Global number of years lived with disability (YLDs) and YLDs due to Guillain–Barre syndrome per 100,000 population attributable to each underlying cause by age in 2019. (Generated from data available from http://ghdx.healthdata.org/gbd-results-tool)

Although the proportion of the GBS YLDs attributable to the individual underlying causes differed by age group, globally upper respiratory infections and unknown causes accounted for most of the YLDs. The highest proportion of attributable YLDs were in the 5–9 age group for all GBS causes. Furthermore, the global YLD rate of GBS attributable to all causes, especially unknown causes and upper respiratory infections, increased with advancing age, except for the Zika virus (Fig. 5). The proportion of GBS prevalence attributable to the individual underlying causes showed a similar pattern to the YLDs by age group (Additional file 12: Fig. S9).

## Discussion

In this study we reported the levels and trends in the global burden of GBS from 1990 to 2019, making comparisons across different regions and countries. Our analyses were based on the 2019 dataset provided by the GBD project, which is the most comprehensive worldwide observational epidemiological study aimed at reporting mortality and disability from major diseases, at the global, national and regional levels [14]. We found a worldwide increase of 6.4% in the age-standardised prevalence of GBS from 1990 to 2019. This slight increase in the age-standardised prevalence may partly reflect the increased life expectancy among GBS patients, due to improved care and earlier diagnosis of this condition [4]. The observed increase in the prevalent cases of GBS can be explained by population growth and aging. However, future studies using decomposition analysis are needed to clarify the roles that population growth and aging had on the number of prevalent cases of GBS. Furthermore, higher exposure to potential underlying causes of GBS, such as infection with *Campylobacter jejuni*, *Haemophilus influenzae*, Cytomegalovirus, Zika virus, and some other respiratory or gastrointestinal infections could be another reason for the observed increase [1, 15]. Nevertheless, future research is required to determine the probable causes of the increased prevalence of GBS over the period 1990–2019.

For both sexes, the prevalence rate of GBS was bimodal, with the highest age-standardised values among the elderly and the lowest in children aged < 4 years. Nevertheless, the absolute number of prevalent GBS cases was highest in children aged 5–9 years, highlighting the need for more health resource allocation and efforts to improve surveillance systems for children, especially for those with underlying causes of GBS. In addition, a “ceiling effect” was found in the age-standardised prevalence rates among women, with the prevalence rate remaining stable after the age of 70. This finding could be attributed to changes of the immune system of women after the menopause [16].

The age-standardised prevalence rates of GBS in 2019 varied considerably by geographic region. The highest age-standardised prevalence rates were observed in High-income Asia Pacific and High-income North America, while the lowest were found in East Asia and Oceania. This variation in the burden of GBS between different regions may be as a result of differing levels of hygiene and variations in local exposure to the risk factors, especially gastrointestinal and respiratory infections [17]. Interestingly, there was considerable variation in the age-standardised prevalence rates of GBS within the countries that comprise Tropical Latin America. Although exposure to the Zika virus was recently identified as an arbovirus infection associated with GBS [18, 19], there is insufficient epidemiological data in the GBD database to conclusively explain these differences.

The highest age-standardised prevalence rate of GBS was found in Japan, probably due to the high frequency of *Campylobacter jejuni* infections and presumably also due to genetic and environmental predispositions [20–22]. The percentage increase in the age-standardised prevalence rate of GBS from 1990 to 2019 in different regions and countries may be due to the recent outbreak of the Zika virus in several countries from the Western Sub-Saharan Africa, Central Latin America and Caribbean regions, such as the Virgin Islands, the USA, Dominica and Puerto Rico. Strategies aimed at controlling the spread of the Zika virus can effectively reduce the burden of GBS in these countries. Although Brazil was one of the countries which was most severely affected by the Zika virus epidemic [23, 24], the age-standardised prevalence rate of GBS decreased significantly (by 40.7%) between 1990 and 2019, due to reductions in cases from other causes. Again it is worth mentioning that, there is insufficient epidemiological data in the GBD database to conclusively explain these differences.

Between 1990 and 2019, GBS was associated with a worldwide increase in both absolute numbers and the age-standardised rate of YLDs, while also being consistently higher in males. Worldwide, GBS was associated with a substantial burden in terms of disability, but there were also regional differences in the GBS-associated disability, the reasons for which should be identified and used to implement specific geographical strategies to improve the health outcomes of people with GBS. Despite the lack of a clear association between development level (regional or country level) and the GBS burden, an observed high in YLDs was found in the High-income Asia Pacific and High-income North American countries. Although the GBD findings could not determine the reasons for higher prevalence of GBS in two mentioned regions, further studies on burden of GBS by type and its attributable underlying causes are recommended.

### Strengths and limitations of the study

Our study is one of the first and most comprehensive epidemiological studies to provide an insight into the burden of GBS at the global, regional and national levels, as well as its trend over the period 1990 to 2019. However, despite its high methodological rigor, the present study is not without limitations. The largest shortcoming is the dearth of data and reliable information for many countries, especially those in developing countries. In resource-limited settings, diagnostic codes may not be entirely accurate, resulting in an underestimation of GBS cases. In these situations, missing data were inferred using statistical approaches by performing covariate-driven modeling. This warrants further action and research in the field, such as establishing ad hoc registries and pursuing nationwide community-based epidemiological surveys. Moreover, the data used to estimate the burden of GBS was based on hospital- and self-report data, which can lead to a high degree of variability and overestimation of the disease burden, as GBS patients who are quite debilitated in hospital may recover significantly. It is also important to recognise that severe cases of GBS in resource limited countries may die prior to getting to a hospital, resulting in a selection bias of less severe cases in the inpatient hospital data. Moreover, the same disability index of 0.296 was used in all countries, GBS disease causes, and ages. This could be inaccurate, as the survival, disability and recovery associated with GBS could vary significantly from country to country, by: level of socioeconomic development, access to tertiary care hospitals with GBS management expertise, proportion of patients with different variants of GBS, and the age of patients with GBS and the proportion with other comorbidities. Similarly, attributing the same disability weight to different causes may not be accurate, as it is known that certain causes (e.g., *Campylobacter jejuni* gastroenteritis) may be associated with higher disability and mortality rates. In addition, the burden of the different subtypes of GBS, such as acute inflammatory demyelinating polyradiculoneuropathy (AIDP), acute motor axonal neuropathy (AMAN) and acute motor sensory axonal neuropathy (AMSAN), were not reported in this study [26]. Due to variations in the outcomes of different GBS variants, providing data on the burden of different GBS variants might be useful for health policy makers and should be taken into account in future research. Furthermore, no data was available for the burden of GBS in some countries and this must be taken into account when interpreting the results of this study.

## Conclusion

The present study examined trends in the burden of GBS from 1990 to 2019 across different regions and countries and found a large burden, in terms of the worldwide prevalence and disability, especially among young children and the elderly. Strategies aimed at preventing infectious diseases and improving hygiene conditions may be effective in reducing the prevalence of GBS. Furthermore, the effects of emergent infectious diseases, such as coronavirus disease 2019 (COVID-19), on the incidence of GBS should be evaluated in future research [27, 28]. Efforts to improve data collection and sharing, especially in low SDI countries, and the implementations of different disability weights for each location and underlying cause for estimation of the burden of GBS should be taken into account in future studies. Finally, geographic differences in the burden of GBS should be considered in future health-related decision-making and planning processes. In addition, the epidemiology of anti-ganglioside antibodies, as one of the associated factors with GBS, is recommended to be discussed in future epidemiological research.

## Supplementary Information

**Additional file 1: Table S1.** Sequelae for Guillain–Barre syndrome and the corresponding disability weights in the GBD 2019 study.

**Additional file 2: Table S2.** Total number of Guillain–Barre syndrome cases in 1990 and 2019 and the percentage change in the age-standardised rates (ASRs) per 100,000, by location (Generated from data available from http://ghdx.healthdata.org/gbd-results-tool).

**Additional file 3: Table S3.** Years lived with disability (YLDs) due to Guillain–Barre syndrome in 1990 and 2019 and the percentage change in the age-standardised rates (ASRs) per 100,000, by location (Generated from data available from http://ghdx.healthdata.org/gbd-results-tool).

**Additional file 4: Figure S1.** The age-standardised point prevalence of Guillain–Barre syndrome in 2019 for the 21 Global Burden of Disease regions, by sex. The error bars represent 95% uncertainty intervals for the age-standardised prevalence per 100,000 population. (Generated from data available from http://ghdx.healthdata.org/gbd-results-tool).

**Additional file 5: Figure S2.** The age-standardised years lived with disability (YLDs) rates of Guillain–Barre syndrome in 2019 for the 21 Global Burden of Disease regions, by sex. The error bars represent 95% uncertainty intervals for the age-standardised YLD rate per 100,000 population. (Generated from data available from http://ghdx.healthdata.org/gbd-results-tool).

**Additional file 6: Figure S3.** The percentage change in the age-standardised point prevalence of Guillain–Barre syndrome from 1990 to 2019 for the 21 Global Burden of Disease regions, by sex. The error bars represent 95% uncertainty intervals for the percentage change in age-standardised prevalence per 100,000 population between 1990 and 2019. (Generated from data available from http://ghdx.healthdata.org/gbd-results-tool).

**Additional file 7: Figure S4.** The percentage change in the age-standardised years lived with disability (YLDs) rates of Guillain–Barre syndrome from 1990 to 2019 for the 21 Global Burden of Disease regions, by sex. The error bars represent 95% uncertainty intervals for the percentage change in age-standardised YLD rates per 100,000 population between 1990 and 2019. (Generated from data available from http://ghdx.healthdata.org/gbd-results-tool).

**Additional file 8: Figure S5.** Global number of years lived with disability (YLDs) cases and years lived with disability (YLDs) of Guillain–Barre syndrome per 100,000 population, by age and sex in 2019. The error bars represent 95% uncertainty intervals for total number of YLDs. Dotted and dashed lines indicate 95% uncertainty intervals for YLD (per 100,000). (Generated from data available from http://ghdx.healthdata.org/gbd-results-tool).

**Additional file 9: Figure S6.** Age-standardised prevalence rates of Guillain–Barre syndrome for 204 countries and territories, by Socio-demographic Index (SDI), in 2019; Expected values based on the Socio-demographic Index and disease rates in all locations are shown as the black line. Each point shows the observed age-standardised YLD rate for each country in 2019. (Generated from data available from http://ghdx.healthdata.org/gbd-results-tool).

**Additional file 10: Figure S7.** Age-standardised prevalence rates of Guillain–Barre syndrome, by Socio-demographic Index (SDI) quintiles, from 1990 to 2019; (Generated from data available from http://ghdx.healthdata.org/gbd-results-tool).

**Additional file 11: Figure S8.** Age-standardised years lived with disability (YLDs) rates of Guillain–Barre syndrome, by Socio-demographic Index (SDI) quintiles, from 1990 to 2019; (Generated from data available from http://ghdx.healthdata.org/gbd-results-tool).

**Additional file 12: Figure S9.** Global total number of cases and prevalence due to Guillain–Barre syndrome per 100,000 population attributable to each underlying cause, by age in 2019. (Generated from data available from http://ghdx.healthdata.org/gbd-results-tool).

## Data Availability

Publicly available datasets were analysed in this study. This data can be found here: http://ghdx.healthdata.org/gbd-results-tool.

## Abbreviations

GBS: Guillain–Barré syndrome
SDI: Socio-demographic Index
GBD: Global burden of diseases
IHME: Institute for Health Metrics and Evaluation
ICD: International Classification of Disease
USA: United States of America
YLD: Years lived with disability
UI: Uncertainty intervals
AIDP: Acute inflammatory demyelinating polyradiculoneuropathy
AMAN: Acute motor axonal neuropathy
AMSAN: Acute motor sensory axonal neuropathy
COVID-19: Coronavirus disease 2019
