# Supplementary material for: Global, regional, and national burden of Guillain–Barré syndrome and its underlying causes from 1990 to 2019
Source: J Neuroinflammation. 2021 Nov 11;18:264. doi: 10.1186/s12974-021-02319-4 (PMC8581128; doi:10.1186/s12974-021-02319-4)
Supplement: Supplementary file 1 — Additional file 1: Table S1. Sequelae for Guillain–Barre syndrome and the corresponding disability weights in the GBD 2019 study. [file 12974_2021_2319_MOESM1_ESM.doc]

| **Table S1: Sequelae for Guillain-Barre syndrome and the corresponding disability weights in the GBD 2019 study** | | |
| --- | --- | --- |
| **Health state** | **Lay description** | **DW**  **(95% CI)** |
| Guillain-Barré syndrome due to lower respiratory infections | This person is paralyzed from the waist down, cannot feel or move the legs and has  difficulties with urine and bowel control. The person uses a wheelchair to move  around. | 0.296 (0.198-0.414) |
| Guillain-Barré syndrome due to upper respiratory infections | This person is paralyzed from the waist down, cannot feel or move the legs and has  difficulties with urine and bowel control. The person uses a wheelchair to move  around. | 0.296  (0.198-0.414) |
| Guillain-Barré syndrome due to diarrheal diseases | This person is paralyzed from the waist down, cannot feel or move the legs and has  difficulties with urine and bowel control. The person uses a wheelchair to move  around. | 0.296  (0.198-0.414) |
| Guillain-Barré syndrome due to other infectious diseases | This person is paralyzed from the waist down, cannot feel or move the legs and has  difficulties with urine and bowel control. The person uses a wheelchair to move  around. | 0.296  (0.198-0.414) |
| **GBD: Global Burden of Disease**  **DW: Disability weight** | | |
