# Supplementary material for: Global, regional, and national burden of Guillain–Barré syndrome and its underlying causes from 1990 to 2019
Source: J Neuroinflammation. 2021 Nov 11;18:264. doi: 10.1186/s12974-021-02319-4 (PMC8581128; doi:10.1186/s12974-021-02319-4)
Supplement: Supplementary file 2 — Additional file 2: Table S2. Total number of Guillain–Barre syndrome cases in 1990 and 2019 and the percentage change in the age-standardised rates (ASRs) per 100,000, by location (Generated from data available from http://ghdx.healthdata.org/gbd-results-tool). [file 12974_2021_2319_MOESM2_ESM.doc]

| **Table S2: Total number of Guillain-Barre syndrome cases in 1990 and 2019 and the percentage change in the age-standardised rates (ASRs) per 100,000, by location**  **(Generated from data available from http://ghdx.healthdata.org/gbd-results-tool)** | | | | | |
| --- | --- | --- | --- | --- | --- |
|  | **1990** | | **2019** | | **Percentage change in ASRs per 100,000** |
|  | **No (95% UI)** | **ASRs per 100,000 (95% UI)** | **No (95% UI)** | **ASRs per 100,000 (95% UI)** |
| **Global** | **90249 (70747 , 114487)** | **1.8 (1.4 , 2.3)** | **150095 (119924 , 188309)** | **1.9 (1.5 , 2.4)** | **6.4 (3.6 , 9.5)** |
| **High-income North America** | **9103 (7253 , 11404)** | **3 (2.4 , 3.8)** | **20834 (16851 , 25728)** | **4.2 (3.5 , 5.1)** | **41.2 (28.8 , 56.1)** |
| **Canada** | **498 (385 , 641)** | **1.7 (1.3 , 2.1)** | **1273 (950 , 1670)** | **2.5 (1.9 , 3.2)** | **50.2 (34.5 , 69.1)** |
| **Greenland** | **1 (1 , 2)** | **2.9 (2.2 , 3.7)** | **2 (1 , 2)** | **2.9 (2.2 , 3.7)** | **1 (0.5 , 1.5)** |
| **United States of America** | **8604 (6828 , 10789)** | **3.2 (2.5 , 4)** | **19559 (15809 , 24066)** | **4.5 (3.7 , 5.4)** | **41.2 (27.4 , 57.3)** |
| **Australasia** | **300 (231 , 386)** | **1.4 (1.1 , 1.8)** | **560 (431 , 726)** | **1.6 (1.3 , 2.1)** | **13.8 (5 , 24.1)** |
| **Australia** | **217 (165 , 284)** | **1.2 (0.9 , 1.6)** | **449 (338 , 592)** | **1.5 (1.2 , 2)** | **24.2 (10.7 , 41.5)** |
| **New Zealand** | **83 (66 , 105)** | **2.4 (1.9 , 3)** | **112 (90 , 137)** | **2.1 (1.7 , 2.5)** | **-12 (-21.9 , -0.6)** |
| **High-income Asia Pacific** | **10146 (8097 , 12606)** | **5.8 (4.6 , 7.2)** | **11708 (9631 , 14284)** | **6.4 (5.2 , 7.7)** | **9.3 (4 , 14.8)** |
| **Brunei Darussalam** | **16 (13 , 21)** | **6.3 (5 , 7.9)** | **28 (22 , 35)** | **6.3 (5 , 7.8)** | **-0.4 (-0.6 , -0.2)** |
| **Japan** | **7104 (5677 , 8827)** | **5.7 (4.5 , 7)** | **7928 (6598 , 9556)** | **6.4 (5.3 , 7.7)** | **12.7 (4.3 , 21.6)** |
| **Singapore** | **194 (153 , 246)** | **6.3 (5 , 7.8)** | **360 (286 , 459)** | **6.3 (5 , 7.8)** | **0.3 (0.1 , 0.6)** |
| **Republic of Korea** | **2831 (2242 , 3551)** | **6.3 (5 , 7.8)** | **3392 (2691 , 4334)** | **6.3 (5 , 7.8)** | **0.4 (0.2 , 0.7)** |
| **Western Europe** | **7262 (5638 , 9322)** | **1.6 (1.3 , 2.1)** | **11188 (8605 , 14514)** | **1.9 (1.5 , 2.4)** | **17 (14.1 , 20.6)** |
| **Andorra** | **1 (1 , 1)** | **1.8 (1.4 , 2.3)** | **2 (1 , 3)** | **1.8 (1.4 , 2.3)** | **-0.4 (-0.7 , -0.2)** |
| **Austria** | **185 (153 , 226)** | **2 (1.6 , 2.4)** | **382 (298 , 488)** | **3 (2.4 , 3.8)** | **53.5 (32.4 , 83)** |
| **Belgium** | **212 (163 , 276)** | **1.8 (1.4 , 2.3)** | **266 (202 , 347)** | **1.8 (1.4 , 2.3)** | **0.3 (0.1 , 0.6)** |
| **Cyprus** | **15 (11 , 19)** | **1.8 (1.4 , 2.3)** | **28 (22 , 37)** | **1.8 (1.4 , 2.3)** | **-0.2 (-0.4 , 0.1)** |
| **Denmark** | **95 (72 , 125)** | **1.5 (1.2 , 2)** | **120 (89 , 161)** | **1.6 (1.2 , 2.1)** | **2.1 (-4.2 , 9.8)** |
| **Finland** | **74 (55 , 98)** | **1.3 (1 , 1.7)** | **119 (88 , 162)** | **1.6 (1.2 , 2.1)** | **23.4 (14 , 35.4)** |
| **France** | **1235 (958 , 1604)** | **1.9 (1.5 , 2.4)** | **1607 (1216 , 2108)** | **1.9 (1.5 , 2.4)** | **0.2 (0 , 0.4)** |
| **Germany** | **1925 (1488 , 2501)** | **2 (1.6 , 2.6)** | **2330 (1762 , 3027)** | **2 (1.6 , 2.6)** | **0.8 (0.4 , 1.3)** |
| **Greece** | **157 (116 , 210)** | **1.3 (1 , 1.7)** | **226 (168 , 302)** | **1.6 (1.2 , 2.1)** | **22.6 (11.9 , 38.2)** |
| **Iceland** | **5 (4 , 6)** | **1.8 (1.4 , 2.3)** | **7 (6 , 10)** | **1.8 (1.4 , 2.3)** | **0.3 (0.2 , 0.5)** |
| **Ireland** | **67 (53 , 86)** | **1.8 (1.4 , 2.3)** | **105 (81 , 138)** | **1.8 (1.4 , 2.3)** | **0.1 (-0.1 , 0.3)** |
| **Israel** | **87 (68 , 111)** | **1.8 (1.4 , 2.3)** | **178 (140 , 229)** | **1.8 (1.4 , 2.3)** | **0.3 (0.2 , 0.5)** |
| **Italy** | **1226 (965 , 1554)** | **2 (1.6 , 2.5)** | **2103 (1669 , 2669)** | **2.3 (1.9 , 2.9)** | **17 (5.6 , 32.1)** |
| **Luxembourg** | **8 (6 , 10)** | **1.8 (1.4 , 2.3)** | **14 (10 , 18)** | **1.8 (1.4 , 2.3)** | **0.7 (0.4 , 1.3)** |
| **Malta** | **7 (6 , 9)** | **1.8 (1.4 , 2.3)** | **11 (8 , 14)** | **1.8 (1.4 , 2.3)** | **0.7 (0.5 , 1)** |
| **Monaco** | **1 (1 , 1)** | **1.8 (1.4 , 2.3)** | **1 (1 , 1)** | **1.8 (1.4 , 2.3)** | **0.3 (0.1 , 0.6)** |
| **Netherlands** | **238 (179 , 314)** | **1.4 (1.1 , 1.9)** | **401 (302 , 541)** | **1.8 (1.4 , 2.3)** | **23.7 (13.5 , 38.7)** |
| **Norway** | **137 (108 , 175)** | **2.8 (2.3 , 3.6)** | **184 (143 , 234)** | **2.9 (2.3 , 3.6)** | **0.6 (0.3 , 0.9)** |
| **Portugal** | **222 (172 , 289)** | **1.9 (1.5 , 2.5)** | **294 (225 , 387)** | **2.1 (1.6 , 2.6)** | **7.3 (1.2 , 14.1)** |
| **San Marino** | **0 (0 , 1)** | **1.8 (1.4 , 2.3)** | **1 (1 , 1)** | **1.8 (1.4 , 2.3)** | **-0.5 (-1 , 0)** |
| **Spain** | **584 (440 , 768)** | **1.3 (1 , 1.7)** | **972 (721 , 1298)** | **1.6 (1.2 , 2.1)** | **17.9 (9.1 , 29.8)** |
| **Sweden** | **116 (88 , 150)** | **1.2 (0.9 , 1.5)** | **300 (226 , 399)** | **2.3 (1.8 , 3)** | **102.6 (85.8 , 122.4)** |
| **Switzerland** | **144 (111 , 187)** | **1.8 (1.4 , 2.3)** | **206 (156 , 270)** | **1.8 (1.4 , 2.3)** | **0.5 (0.3 , 0.8)** |
| **United Kingdom** | **514 (382 , 680)** | **0.8 (0.6 , 1)** | **1321 (991 , 1761)** | **1.6 (1.2 , 2)** | **104.7 (90.1 , 124.4)** |
| **Southern Latin America** | **1214 (946 , 1548)** | **2.5 (1.9 , 3.2)** | **1764 (1371 , 2270)** | **2.5 (1.9 , 3.2)** | **0.1 (0.1 , 0.2)** |
| **Argentina** | **826 (646 , 1058)** | **2.5 (1.9 , 3.2)** | **1174 (915 , 1499)** | **2.5 (1.9 , 3.2)** | **0.1 (0 , 0.2)** |
| **Chile** | **304 (236 , 391)** | **2.5 (1.9 , 3.2)** | **493 (383 , 641)** | **2.5 (1.9 , 3.2)** | **0.2 (0.1 , 0.2)** |
| **Uruguay** | **84 (65 , 107)** | **2.5 (1.9 , 3.2)** | **97 (75 , 125)** | **2.5 (1.9 , 3.2)** | **-0.1 (-0.1 , 0)** |
| **Eastern Europe** | **4419 (3436 , 5641)** | **1.9 (1.5 , 2.4)** | **4323 (3349 , 5635)** | **1.9 (1.5 , 2.4)** | **0.4 (0.2 , 0.6)** |
| **Belarus** | **191 (147 , 242)** | **1.8 (1.4 , 2.2)** | **185 (141 , 240)** | **1.8 (1.4 , 2.2)** | **0.1 (0 , 0.3)** |
| **Estonia** | **29 (22 , 37)** | **1.8 (1.4 , 2.2)** | **26 (20 , 34)** | **1.8 (1.4 , 2.2)** | **0.2 (0 , 0.5)** |
| **Latvia** | **49 (38 , 62)** | **1.8 (1.4 , 2.2)** | **39 (30 , 50)** | **1.8 (1.4 , 2.2)** | **0.2 (0 , 0.4)** |
| **Lithuania** | **67 (52 , 85)** | **1.8 (1.4 , 2.2)** | **56 (43 , 73)** | **1.8 (1.4 , 2.2)** | **0 (-0.1 , 0.2)** |
| **Republic of Moldova** | **78 (60 , 100)** | **1.8 (1.4 , 2.2)** | **71 (54 , 91)** | **1.8 (1.4 , 2.2)** | **0.1 (0 , 0.2)** |
| **Russian Federation** | **2951 (2301 , 3772)** | **1.9 (1.5 , 2.4)** | **3027 (2350 , 3944)** | **1.9 (1.5 , 2.4)** | **0.3 (0.1 , 0.6)** |
| **Ukraine** | **1053 (818 , 1353)** | **1.9 (1.5 , 2.4)** | **919 (713 , 1196)** | **1.9 (1.5 , 2.4)** | **0.2 (0.1 , 0.4)** |
| **Central Europe** | **1837 (1402 , 2391)** | **1.4 (1.1 , 1.9)** | **1858 (1424 , 2429)** | **1.4 (1.1 , 1.8)** | **-1.9 (-5.6 , 2.7)** |
| **Albania** | **44 (34 , 57)** | **1.4 (1.1 , 1.8)** | **43 (33 , 56)** | **1.4 (1.1 , 1.8)** | **0 (-0.3 , 0.3)** |
| **Bosnia and Herzegovina** | **61 (47 , 79)** | **1.4 (1.1 , 1.8)** | **52 (39 , 70)** | **1.4 (1.1 , 1.8)** | **0.2 (0 , 0.4)** |
| **Bulgaria** | **134 (103 , 176)** | **1.4 (1.1 , 1.8)** | **116 (87 , 155)** | **1.4 (1.1 , 1.8)** | **-0.1 (-0.3 , 0)** |
| **Croatia** | **73 (56 , 95)** | **1.4 (1.1 , 1.8)** | **70 (53 , 94)** | **1.4 (1.1 , 1.8)** | **0.4 (0.1 , 0.7)** |
| **Czechia** | **154 (118 , 199)** | **1.4 (1.1 , 1.8)** | **175 (133 , 232)** | **1.4 (1.1 , 1.8)** | **0.4 (0.2 , 0.7)** |
| **Hungary** | **158 (121 , 205)** | **1.4 (1.1 , 1.8)** | **158 (120 , 211)** | **1.4 (1.1 , 1.8)** | **0.1 (0 , 0.2)** |
| **Montenegro** | **9 (7 , 12)** | **1.4 (1.1 , 1.9)** | **10 (8 , 13)** | **1.4 (1.1 , 1.9)** | **0.2 (0.1 , 0.4)** |
| **North Macedonia** | **28 (22 , 37)** | **1.4 (1.1 , 1.8)** | **33 (26 , 44)** | **1.4 (1.1 , 1.8)** | **0 (-0.1 , 0.2)** |
| **Poland** | **603 (452 , 805)** | **1.6 (1.2 , 2)** | **641 (506 , 812)** | **1.5 (1.2 , 1.8)** | **-5.8 (-15.9 , 7.6)** |
| **Romania** | **346 (265 , 444)** | **1.4 (1.1 , 1.8)** | **314 (240 , 416)** | **1.4 (1.1 , 1.8)** | **0 (-0.1 , 0.1)** |
| **Serbia** | **122 (92 , 160)** | **1.2 (0.9 , 1.6)** | **125 (94 , 168)** | **1.2 (0.9 , 1.6)** | **0.4 (0.2 , 0.7)** |
| **Slovakia** | **77 (59 , 99)** | **1.4 (1.1 , 1.8)** | **86 (66 , 115)** | **1.4 (1.1 , 1.8)** | **0.1 (0 , 0.3)** |
| **Slovenia** | **29 (22 , 37)** | **1.4 (1.1 , 1.8)** | **34 (26 , 46)** | **1.4 (1.1 , 1.8)** | **0.6 (0.2 , 1.1)** |
| **Central Asia** | **1205 (938 , 1506)** | **1.9 (1.5 , 2.4)** | **1695 (1314 , 2135)** | **1.9 (1.5 , 2.4)** | **0 (-0.7 , 0.6)** |
| **Armenia** | **61 (48 , 78)** | **1.9 (1.5 , 2.4)** | **62 (48 , 79)** | **1.9 (1.5 , 2.4)** | **0.3 (0.2 , 0.5)** |
| **Azerbaijan** | **127 (99 , 160)** | **1.9 (1.5 , 2.4)** | **193 (149 , 247)** | **1.9 (1.5 , 2.4)** | **0.4 (0.1 , 0.7)** |
| **Georgia** | **107 (83 , 137)** | **1.9 (1.5 , 2.4)** | **73 (59 , 91)** | **1.7 (1.4 , 2.1)** | **-7.4 (-17.5 , 5.2)** |
| **Kazakhstan** | **292 (227 , 367)** | **1.9 (1.5 , 2.4)** | **346 (269 , 438)** | **1.9 (1.5 , 2.4)** | **0.1 (-0.1 , 0.3)** |
| **Kyrgyzstan** | **78 (60 , 98)** | **1.9 (1.5 , 2.4)** | **116 (90 , 146)** | **1.9 (1.5 , 2.4)** | **0.3 (0.1 , 0.6)** |
| **Mongolia** | **36 (28 , 45)** | **1.9 (1.5 , 2.4)** | **60 (47 , 77)** | **1.9 (1.5 , 2.4)** | **0.3 (0.1 , 0.5)** |
| **Tajikistan** | **90 (70 , 114)** | **1.9 (1.5 , 2.4)** | **162 (125 , 204)** | **1.9 (1.5 , 2.4)** | **0.4 (0.1 , 0.7)** |
| **Turkmenistan** | **62 (48 , 78)** | **1.9 (1.5 , 2.4)** | **92 (71 , 116)** | **1.9 (1.5 , 2.4)** | **0.2 (-0.1 , 0.5)** |
| **Uzbekistan** | **352 (272 , 444)** | **1.9 (1.5 , 2.4)** | **590 (455 , 749)** | **1.9 (1.5 , 2.4)** | **0.3 (0.1 , 0.5)** |
| **Central Latin America** | **5660 (4324 , 7253)** | **3.8 (3 , 4.8)** | **9501 (7587 , 11892)** | **3.9 (3.1 , 4.9)** | **2.7 (-0.9 , 7.1)** |
| **Colombia** | **1041 (802 , 1322)** | **3.5 (2.8 , 4.5)** | **1690 (1342 , 2142)** | **3.5 (2.8 , 4.5)** | **-0.2 (-0.4 , 0)** |
| **Costa Rica** | **99 (76 , 126)** | **3.6 (2.8 , 4.5)** | **166 (131 , 211)** | **3.5 (2.8 , 4.5)** | **-0.3 (-0.6 , -0.1)** |
| **El Salvador** | **157 (120 , 201)** | **3.2 (2.6 , 4.1)** | **202 (159 , 251)** | **3.3 (2.6 , 4.1)** | **2.3 (-4 , 9.7)** |
| **Guatemala** | **264 (199 , 345)** | **3.6 (2.8 , 4.5)** | **571 (447 , 722)** | **3.5 (2.8 , 4.4)** | **-0.7 (-1.1 , -0.1)** |
| **Honduras** | **136 (102 , 180)** | **3.2 (2.5 , 4)** | **293 (228 , 376)** | **3.3 (2.6 , 4.2)** | **2.8 (-3.7 , 10.7)** |
| **Mexico** | **3154 (2384 , 4098)** | **4.1 (3.2 , 5.2)** | **5218 (4174 , 6511)** | **4.3 (3.4 , 5.3)** | **5.3 (-1.3 , 14.1)** |
| **Nicaragua** | **127 (95 , 168)** | **3.5 (2.8 , 4.4)** | **213 (168 , 270)** | **3.5 (2.8 , 4.5)** | **0.5 (0.1 , 1)** |
| **Panama** | **78 (61 , 99)** | **3.6 (2.8 , 4.5)** | **151 (119 , 191)** | **3.7 (2.9 , 4.6)** | **2.3 (0.7 , 5.9)** |
| **Venezuela (Bolivarian Republic of)** | **603 (466 , 765)** | **3.5 (2.8 , 4.5)** | **996 (782 , 1263)** | **3.5 (2.8 , 4.4)** | **-0.2 (-0.3 , -0.1)** |
| **Andean Latin America** | **751 (598 , 931)** | **2.4 (1.9 , 3)** | **1373 (1096 , 1717)** | **2.3 (1.8 , 2.8)** | **-6.8 (-8.6 , -4.7)** |
| **Bolivia (Plurinational State of)** | **122 (96 , 155)** | **2.4 (1.9 , 3.1)** | **261 (206 , 333)** | **2.4 (1.9 , 3.1)** | **1 (0.6 , 2.2)** |
| **Ecuador** | **201 (164 , 241)** | **2.5 (2.1 , 3)** | **305 (249 , 370)** | **1.8 (1.5 , 2.2)** | **-26.1 (-32 , -19)** |
| **Peru** | **428 (334 , 543)** | **2.4 (1.9 , 3.1)** | **808 (637 , 1030)** | **2.4 (1.9 , 3.1)** | **0.6 (0.1 , 1.9)** |
| **Caribbean** | **673 (524 , 856)** | **2 (1.6 , 2.6)** | **980 (771 , 1248)** | **2 (1.6 , 2.6)** | **0 (-0.1 , 0.1)** |
| **Antigua and Barbuda** | **1 (1 , 1)** | **2 (1.6 , 2.6)** | **2 (1 , 2)** | **2 (1.6 , 2.6)** | **0.5 (0.3 , 0.8)** |
| **Barbados** | **5 (4 , 7)** | **2 (1.6 , 2.6)** | **7 (5 , 9)** | **2 (1.6 , 2.6)** | **0.6 (0.3 , 1.1)** |
| **Belize** | **3 (3 , 4)** | **2.1 (1.6 , 2.6)** | **8 (6 , 10)** | **2.1 (1.6 , 2.6)** | **1.8 (0.6 , 4.6)** |
| **Bermuda** | **1 (1 , 2)** | **2 (1.6 , 2.6)** | **2 (1 , 2)** | **2 (1.6 , 2.6)** | **0.3 (0.1 , 0.4)** |
| **Bahamas** | **5 (4 , 6)** | **2 (1.6 , 2.6)** | **8 (6 , 10)** | **2 (1.6 , 2.6)** | **0.2 (0.1 , 0.4)** |
| **Cuba** | **215 (169 , 272)** | **2 (1.6 , 2.6)** | **266 (206 , 347)** | **2 (1.6 , 2.6)** | **0 (-0.2 , 0.2)** |
| **Dominica** | **1 (1 , 2)** | **2 (1.6 , 2.6)** | **1 (1 , 2)** | **2 (1.6 , 2.6)** | **0.9 (0.3 , 1.5)** |
| **Dominican Republic** | **131 (101 , 166)** | **2 (1.6 , 2.6)** | **214 (168 , 273)** | **2 (1.6 , 2.6)** | **0.2 (0 , 0.4)** |
| **Grenada** | **2 (1 , 2)** | **2 (1.6 , 2.6)** | **2 (2 , 3)** | **2 (1.6 , 2.6)** | **0.5 (0.2 , 1)** |
| **Guyana** | **14 (11 , 18)** | **2 (1.6 , 2.6)** | **15 (12 , 19)** | **2 (1.6 , 2.6)** | **-0.2 (-0.3 , -0.1)** |
| **Haiti** | **117 (89 , 150)** | **2 (1.6 , 2.6)** | **228 (176 , 289)** | **2 (1.6 , 2.6)** | **0 (-0.2 , 0.3)** |
| **Jamaica** | **45 (35 , 57)** | **2 (1.6 , 2.6)** | **58 (45 , 74)** | **2 (1.6 , 2.6)** | **0.2 (0.1 , 0.4)** |
| **Puerto Rico** | **73 (57 , 93)** | **2 (1.6 , 2.6)** | **85 (66 , 111)** | **2 (1.6 , 2.6)** | **-0.1 (-0.2 , 0)** |
| **Saint Kitts and Nevis** | **1 (1 , 1)** | **2 (1.6 , 2.6)** | **1 (1 , 2)** | **2 (1.6 , 2.6)** | **0.4 (0.2 , 0.7)** |
| **Saint Lucia** | **3 (2 , 3)** | **2 (1.6 , 2.6)** | **4 (3 , 5)** | **2 (1.6 , 2.6)** | **0.6 (0.4 , 0.9)** |
| **Saint Vincent and the Grenadines** | **2 (2 , 3)** | **2 (1.6 , 2.6)** | **2 (2 , 3)** | **2.1 (1.6 , 2.6)** | **0.9 (0.5 , 1.4)** |
| **Suriname** | **7 (6 , 9)** | **2 (1.6 , 2.6)** | **12 (9 , 15)** | **2 (1.6 , 2.6)** | **-0.3 (-0.5 , -0.1)** |
| **Trinidad and Tobago** | **23 (18 , 29)** | **2 (1.6 , 2.6)** | **30 (24 , 39)** | **2 (1.6 , 2.6)** | **0.2 (0.1 , 0.3)** |
| **United States Virgin Islands** | **2 (2 , 3)** | **2 (1.6 , 2.6)** | **2 (2 , 3)** | **2 (1.6 , 2.6)** | **0 (-0.1 , 0.2)** |
| **Tropical Latin America** | **2853 (2143 , 3815)** | **2.3 (1.7 , 3.1)** | **3205 (2530 , 4006)** | **1.4 (1.1 , 1.7)** | **-40.3 (-49.9 , -31.3)** |
| **Brazil** | **2818 (2114 , 3770)** | **2.3 (1.7 , 3.1)** | **3132 (2470 , 3918)** | **1.4 (1.1 , 1.7)** | **-40.7 (-50.4 , -31.6)** |
| **Paraguay** | **36 (27 , 46)** | **1.1 (0.9 , 1.5)** | **73 (54 , 97)** | **1.1 (0.8 , 1.5)** | **0.2 (-5.9 , 7.5)** |
| **East Asia** | **8164 (5975 , 10880)** | **0.7 (0.5 , 0.9)** | **11886 (8933 , 15912)** | **0.8 (0.6 , 1)** | **11.4 (4.7 , 18.7)** |
| **China** | **7802 (5700 , 10413)** | **0.7 (0.5 , 0.9)** | **11313 (8453 , 15144)** | **0.8 (0.6 , 1)** | **11.3 (4.3 , 18.9)** |
| **Democratic People's Republic of Korea** | **169 (126 , 222)** | **0.9 (0.6 , 1.1)** | **226 (170 , 298)** | **0.9 (0.6 , 1.1)** | **0.3 (-0.1 , 0.8)** |
| **Taiwan (Province of China)** | **192 (145 , 250)** | **1 (0.8 , 1.3)** | **347 (265 , 460)** | **1.2 (1 , 1.6)** | **24.1 (11.2 , 51)** |
| **Southeast Asia** | **4791 (3566 , 6249)** | **1.1 (0.8 , 1.4)** | **7087 (5401 , 9186)** | **1.1 (0.8 , 1.4)** | **0.3 (0.1 , 0.6)** |
| **Cambodia** | **100 (72 , 135)** | **1 (0.8 , 1.3)** | **162 (121 , 213)** | **1 (0.8 , 1.3)** | **0.1 (-0.2 , 0.4)** |
| **Indonesia** | **1992 (1483 , 2602)** | **1.1 (0.9 , 1.5)** | **2806 (2121 , 3623)** | **1.1 (0.9 , 1.5)** | **0 (-0.1 , 0.1)** |
| **Lao People's Democratic Republic** | **40 (29 , 54)** | **1 (0.8 , 1.3)** | **69 (51 , 90)** | **1 (0.8 , 1.3)** | **0.2 (0.1 , 0.4)** |
| **Malaysia** | **170 (125 , 225)** | **1 (0.8 , 1.4)** | **310 (234 , 404)** | **1 (0.8 , 1.4)** | **0.1 (0 , 0.3)** |
| **Maldives** | **2 (2 , 3)** | **1 (0.8 , 1.4)** | **5 (3 , 6)** | **1 (0.8 , 1.4)** | **0 (-0.7 , 0.8)** |
| **Mauritius** | **11 (8 , 14)** | **1 (0.8 , 1.3)** | **14 (10 , 18)** | **1 (0.8 , 1.3)** | **0.1 (0 , 0.2)** |
| **Myanmar** | **399 (293 , 527)** | **1 (0.8 , 1.3)** | **541 (408 , 708)** | **1 (0.8 , 1.3)** | **-0.1 (-0.3 , 0)** |
| **Philippines** | **682 (506 , 896)** | **1.1 (0.9 , 1.5)** | **1216 (917 , 1570)** | **1.1 (0.9 , 1.5)** | **-0.2 (-0.4 , 0)** |
| **Sri Lanka** | **167 (124 , 219)** | **1 (0.8 , 1.4)** | **228 (173 , 298)** | **1 (0.8 , 1.3)** | **-0.4 (-0.8 , -0.1)** |
| **Seychelles** | **1 (1 , 1)** | **1 (0.8 , 1.3)** | **1 (1 , 1)** | **1 (0.8 , 1.3)** | **0.3 (0 , 0.5)** |
| **Thailand** | **546 (408 , 717)** | **1 (0.8 , 1.3)** | **755 (574 , 984)** | **1 (0.8 , 1.3)** | **0.1 (0 , 0.1)** |
| **Timor-Leste** | **7 (5 , 10)** | **1 (0.8 , 1.4)** | **13 (10 , 17)** | **1 (0.8 , 1.4)** | **0 (-0.1 , 0.2)** |
| **Viet Nam** | **667 (490 , 886)** | **1 (0.8 , 1.3)** | **959 (726 , 1255)** | **1 (0.8 , 1.3)** | **0.2 (0 , 0.4)** |
| **Oceania** | **62 (45 , 83)** | **1 (0.8 , 1.4)** | **127 (94 , 167)** | **1 (0.8 , 1.4)** | **0 (-0.1 , 0)** |
| **American Samoa** | **0 (0 , 1)** | **1 (0.8 , 1.4)** | **1 (0 , 1)** | **1 (0.8 , 1.3)** | **-0.1 (-0.3 , 0)** |
| **Cook Islands** | **0 (0 , 0)** | **1 (0.8 , 1.4)** | **0 (0 , 0)** | **1 (0.8 , 1.3)** | **-0.2 (-0.6 , 0.1)** |
| **Micronesia (Federated States of)** | **1 (1 , 1)** | **1 (0.8 , 1.3)** | **1 (1 , 1)** | **1 (0.8 , 1.3)** | **-0.1 (-0.3 , 0.1)** |
| **Fiji** | **7 (5 , 10)** | **1 (0.8 , 1.3)** | **9 (7 , 12)** | **1 (0.8 , 1.3)** | **-0.2 (-0.4 , 0)** |
| **Guam** | **1 (1 , 2)** | **1 (0.8 , 1.4)** | **2 (1 , 2)** | **1 (0.8 , 1.3)** | **-0.2 (-0.3 , 0)** |
| **Kiribati** | **1 (1 , 1)** | **1 (0.8 , 1.3)** | **1 (1 , 1)** | **1 (0.8 , 1.3)** | **-0.1 (-0.2 , 0)** |
| **Marshall Islands** | **0 (0 , 1)** | **1 (0.8 , 1.3)** | **1 (0 , 1)** | **1 (0.8 , 1.4)** | **0.4 (0.1 , 0.8)** |
| **Nauru** | **0 (0 , 0)** | **1 (0.8 , 1.4)** | **0 (0 , 0)** | **1 (0.8 , 1.4)** | **-0.1 (-0.6 , 0.3)** |
| **Niue** | **0 (0 , 0)** | **1 (0.8 , 1.3)** | **0 (0 , 0)** | **1 (0.8 , 1.3)** | **0.1 (0 , 0.3)** |
| **Northern Mariana Islands** | **0 (0 , 1)** | **1 (0.8 , 1.4)** | **0 (0 , 1)** | **1 (0.8 , 1.4)** | **-0.3 (-0.8 , 0.3)** |
| **Palau** | **0 (0 , 0)** | **1 (0.8 , 1.3)** | **0 (0 , 0)** | **1 (0.8 , 1.4)** | **0.3 (-0.1 , 0.6)** |
| **Papua New Guinea** | **39 (29 , 52)** | **1 (0.8 , 1.4)** | **94 (69 , 124)** | **1 (0.8 , 1.4)** | **0 (-0.1 , 0.1)** |
| **Samoa** | **2 (1 , 2)** | **1 (0.8 , 1.4)** | **2 (2 , 3)** | **1 (0.8 , 1.4)** | **0.1 (-0.1 , 0.3)** |
| **Solomon Islands** | **3 (2 , 4)** | **1 (0.8 , 1.4)** | **6 (5 , 8)** | **1 (0.8 , 1.4)** | **-0.2 (-0.5 , 0.1)** |
| **Tokelau** | **0 (0 , 0)** | **1 (0.8 , 1.3)** | **0 (0 , 0)** | **1 (0.8 , 1.4)** | **0.3 (-0.1 , 0.7)** |
| **Tonga** | **1 (1 , 1)** | **1 (0.8 , 1.3)** | **1 (1 , 1)** | **1 (0.8 , 1.3)** | **-0.1 (-0.3 , 0)** |
| **Tuvalu** | **0 (0 , 0)** | **1 (0.8 , 1.3)** | **0 (0 , 0)** | **1 (0.8 , 1.4)** | **0.6 (0.1 , 1.2)** |
| **Vanuatu** | **1 (1 , 2)** | **1 (0.8 , 1.4)** | **3 (2 , 4)** | **1 (0.8 , 1.4)** | **-0.2 (-0.3 , 0)** |
| **North Africa and Middle East** | **5284 (4065 , 6783)** | **1.8 (1.4 , 2.3)** | **10386 (8094 , 13324)** | **1.8 (1.4 , 2.3)** | **0.3 (-0.9 , 1.5)** |
| **Afghanistan** | **180 (139 , 232)** | **1.8 (1.4 , 2.3)** | **553 (417 , 723)** | **1.8 (1.4 , 2.3)** | **-0.3 (-0.8 , 0.2)** |
| **Algeria** | **382 (293 , 493)** | **1.8 (1.4 , 2.3)** | **730 (565 , 939)** | **1.8 (1.4 , 2.3)** | **-0.1 (-0.2 , 0.1)** |
| **Bahrain** | **8 (6 , 10)** | **1.8 (1.4 , 2.3)** | **27 (20 , 36)** | **1.8 (1.4 , 2.3)** | **-0.4 (-0.9 , 0)** |
| **Egypt** | **865 (665 , 1112)** | **1.8 (1.4 , 2.3)** | **1632 (1269 , 2102)** | **1.8 (1.4 , 2.3)** | **0 (-0.2 , 0.3)** |
| **Iran (Islamic Republic of)** | **840 (636 , 1086)** | **1.7 (1.3 , 2.2)** | **1424 (1089 , 1857)** | **1.7 (1.3 , 2.2)** | **0.2 (-0.1 , 0.4)** |
| **Iraq** | **261 (200 , 336)** | **1.8 (1.4 , 2.3)** | **669 (518 , 860)** | **1.8 (1.4 , 2.3)** | **0 (0 , 0)** |
| **Jordan** | **63 (48 , 80)** | **2 (1.6 , 2.6)** | **243 (191 , 310)** | **2.3 (1.8 , 2.9)** | **11.4 (3.6 , 20.5)** |
| **Kuwait** | **27 (20 , 35)** | **1.8 (1.4 , 2.3)** | **80 (60 , 108)** | **1.9 (1.5 , 2.4)** | **2 (-4 , 9.2)** |
| **Lebanon** | **54 (42 , 69)** | **1.8 (1.4 , 2.3)** | **94 (73 , 120)** | **1.8 (1.4 , 2.3)** | **0.2 (-0.1 , 0.6)** |
| **Libya** | **47 (36 , 59)** | **1.3 (1.1 , 1.7)** | **106 (80 , 138)** | **1.6 (1.2 , 2.1)** | **19.2 (6.4 , 36.4)** |
| **Morocco** | **392 (303 , 502)** | **1.8 (1.4 , 2.3)** | **640 (495 , 824)** | **1.8 (1.4 , 2.3)** | **0 (-0.1 , 0.1)** |
| **Palestine** | **30 (23 , 39)** | **1.8 (1.4 , 2.3)** | **77 (59 , 99)** | **1.8 (1.4 , 2.3)** | **-0.5 (-1 , -0.1)** |
| **Oman** | **29 (22 , 37)** | **1.8 (1.4 , 2.3)** | **72 (54 , 94)** | **1.8 (1.4 , 2.3)** | **-0.2 (-0.5 , 0.1)** |
| **Qatar** | **7 (5 , 9)** | **1.8 (1.4 , 2.3)** | **47 (34 , 63)** | **1.8 (1.4 , 2.3)** | **0.3 (-0.3 , 1)** |
| **Saudi Arabia** | **236 (179 , 305)** | **1.8 (1.4 , 2.3)** | **599 (446 , 791)** | **1.8 (1.4 , 2.3)** | **0.3 (0.1 , 0.4)** |
| **Sudan** | **301 (231 , 388)** | **1.8 (1.4 , 2.3)** | **622 (477 , 799)** | **1.8 (1.4 , 2.3)** | **0 (-0.1 , 0.2)** |
| **Syrian Arab Republic** | **189 (143 , 245)** | **1.8 (1.4 , 2.3)** | **258 (199 , 333)** | **1.8 (1.4 , 2.3)** | **-0.1 (-0.4 , 0.3)** |
| **Tunisia** | **133 (103 , 171)** | **1.8 (1.4 , 2.3)** | **219 (170 , 284)** | **1.8 (1.4 , 2.3)** | **0 (-0.2 , 0.1)** |
| **Turkey** | **1013 (786 , 1294)** | **1.9 (1.5 , 2.5)** | **1645 (1290 , 2137)** | **1.9 (1.5 , 2.5)** | **0.8 (-5 , 7.6)** |
| **United Arab Emirates** | **28 (20 , 36)** | **1.8 (1.4 , 2.3)** | **165 (114 , 233)** | **1.8 (1.4 , 2.3)** | **-0.1 (-0.6 , 0.4)** |
| **Yemen** | **198 (149 , 258)** | **1.8 (1.4 , 2.3)** | **475 (363 , 610)** | **1.8 (1.4 , 2.3)** | **0.3 (0 , 0.5)** |
| **South Asia** | **16810 (12926 , 21401)** | **1.7 (1.3 , 2.2)** | **30312 (23426 , 38597)** | **1.8 (1.4 , 2.2)** | **1.4 (-2.9 , 5.6)** |
| **Bangladesh** | **1364 (1034 , 1766)** | **1.5 (1.2 , 1.9)** | **2287 (1787 , 2935)** | **1.5 (1.2 , 1.9)** | **-0.1 (-0.4 , 0.3)** |
| **Bhutan** | **8 (6 , 10)** | **1.5 (1.2 , 2)** | **11 (8 , 14)** | **1.5 (1.2 , 1.9)** | **-1.2 (-1.7 , -0.8)** |
| **India** | **13595 (10388 , 17266)** | **1.8 (1.4 , 2.3)** | **24459 (18895 , 30836)** | **1.8 (1.4 , 2.3)** | **2 (-3.5 , 7.3)** |
| **Nepal** | **229 (173 , 295)** | **1.4 (1.1 , 1.8)** | **330 (255 , 416)** | **1.2 (0.9 , 1.5)** | **-15.8 (-22.5 , -7.4)** |
| **Pakistan** | **1614 (1233 , 2058)** | **1.6 (1.3 , 2.1)** | **3226 (2466 , 4157)** | **1.6 (1.3 , 2.1)** | **0.2 (-0.1 , 0.5)** |
| **Southern Sub-Saharan Africa** | **1652 (1284 , 2071)** | **3.4 (2.7 , 4.2)** | **2585 (2032 , 3229)** | **3.4 (2.7 , 4.2)** | **-0.1 (-0.3 , 0)** |
| **Botswana** | **38 (29 , 48)** | **3.2 (2.5 , 4)** | **71 (56 , 90)** | **3.2 (2.5 , 4)** | **0.2 (-0.1 , 0.4)** |
| **Lesotho** | **53 (41 , 68)** | **3.2 (2.5 , 4)** | **63 (49 , 80)** | **3.2 (2.5 , 4)** | **-0.1 (-0.4 , 0.1)** |
| **Namibia** | **41 (32 , 52)** | **3.2 (2.5 , 4)** | **72 (56 , 90)** | **3.2 (2.5 , 4)** | **-0.4 (-0.6 , -0.2)** |
| **South Africa** | **1203 (938 , 1515)** | **3.5 (2.8 , 4.3)** | **1906 (1504 , 2392)** | **3.5 (2.8 , 4.3)** | **0.1 (-0.1 , 0.2)** |
| **Eswatini** | **23 (18 , 29)** | **3.2 (2.5 , 4)** | **34 (26 , 43)** | **3.2 (2.5 , 4)** | **-0.1 (-0.5 , 0.3)** |
| **Zimbabwe** | **295 (228 , 380)** | **3.2 (2.5 , 4)** | **438 (341 , 560)** | **3.2 (2.5 , 4)** | **-0.8 (-1.2 , -0.4)** |
| **Western Sub-Saharan Africa** | **4352 (3350 , 5542)** | **2.5 (2 , 3.2)** | **10306 (7889 , 13113)** | **2.5 (2 , 3.2)** | **-0.4 (-0.9 , 0)** |
| **Benin** | **103 (78 , 132)** | **2.4 (1.9 , 3)** | **270 (205 , 348)** | **2.4 (1.9 , 3)** | **0 (-0.3 , 0.2)** |
| **Burkina Faso** | **205 (156 , 263)** | **2.4 (1.9 , 3)** | **485 (369 , 622)** | **2.4 (1.9 , 3)** | **-0.1 (-0.3 , 0.1)** |
| **Cameroon** | **223 (170 , 284)** | **2.4 (1.9 , 3)** | **629 (479 , 801)** | **2.4 (1.9 , 3)** | **0 (-0.1 , 0.1)** |
| **Cabo Verde** | **8 (6 , 10)** | **2.4 (1.9 , 3)** | **13 (10 , 17)** | **2.4 (1.9 , 3)** | **0.7 (0.1 , 1.5)** |
| **Chad** | **129 (98 , 164)** | **2.4 (1.9 , 3)** | **344 (259 , 447)** | **2.4 (1.9 , 3.1)** | **0.7 (0.3 , 1.1)** |
| **CÃ´te d'Ivoire** | **258 (195 , 334)** | **2.4 (1.9 , 3.1)** | **570 (435 , 724)** | **2.4 (1.9 , 3.1)** | **-0.1 (-0.3 , 0)** |
| **Gambia** | **21 (16 , 27)** | **2.4 (1.9 , 3.1)** | **48 (37 , 62)** | **2.4 (1.9 , 3)** | **-0.4 (-0.7 , -0.1)** |
| **Ghana** | **322 (245 , 413)** | **2.4 (1.9 , 3)** | **698 (532 , 885)** | **2.4 (1.9 , 3)** | **-0.5 (-0.7 , -0.2)** |
| **Guinea** | **135 (104 , 171)** | **2.4 (1.9 , 3)** | **272 (207 , 349)** | **2.4 (1.9 , 3)** | **0 (-0.1 , 0.1)** |
| **Guinea-Bissau** | **21 (16 , 27)** | **2.4 (1.9 , 3)** | **41 (31 , 52)** | **2.4 (1.9 , 3)** | **-0.2 (-0.5 , 0)** |
| **Liberia** | **43 (34 , 55)** | **2.4 (1.9 , 3.1)** | **105 (80 , 133)** | **2.4 (1.9 , 3.1)** | **0 (-0.3 , 0.2)** |
| **Mali** | **187 (144 , 239)** | **2.4 (1.9 , 3)** | **466 (354 , 599)** | **2.4 (1.9 , 3.1)** | **0.3 (0.2 , 0.5)** |
| **Mauritania** | **45 (34 , 57)** | **2.4 (1.9 , 3)** | **89 (68 , 113)** | **2.4 (1.9 , 3.1)** | **0.3 (0.1 , 0.5)** |
| **Niger** | **169 (128 , 217)** | **2.4 (1.9 , 3.1)** | **487 (366 , 634)** | **2.4 (1.9 , 3)** | **-0.2 (-0.5 , 0.1)** |
| **Nigeria** | **2162 (1672 , 2780)** | **2.6 (2.1 , 3.3)** | **5100 (3904 , 6549)** | **2.6 (2.1 , 3.3)** | **-0.8 (-1.6 , -0.1)** |
| **Sao Tome and Principe** | **3 (2 , 3)** | **2.4 (1.9 , 3)** | **5 (3 , 6)** | **2.4 (1.9 , 3)** | **0.3 (0.1 , 0.6)** |
| **Senegal** | **163 (124 , 208)** | **2.4 (1.9 , 3)** | **332 (253 , 422)** | **2.4 (1.9 , 3)** | **-0.1 (-0.3 , 0.1)** |
| **Sierra Leone** | **80 (61 , 101)** | **2.4 (1.9 , 3.1)** | **179 (136 , 228)** | **2.4 (1.9 , 3)** | **0 (-0.2 , 0.1)** |
| **Togo** | **77 (58 , 99)** | **2.4 (1.9 , 3)** | **174 (133 , 221)** | **2.4 (1.9 , 3)** | **-0.4 (-0.8 , -0.2)** |
| **Eastern Sub-Saharan Africa** | **2528 (1885 , 3289)** | **1.5 (1.2 , 1.9)** | **5580 (4169 , 7187)** | **1.5 (1.2 , 2)** | **1.7 (0.5 , 2.9)** |
| **Burundi** | **72 (53 , 94)** | **1.5 (1.1 , 1.9)** | **155 (115 , 201)** | **1.5 (1.1 , 1.9)** | **0.2 (-0.3 , 0.7)** |
| **Comoros** | **6 (5 , 8)** | **1.5 (1.1 , 1.9)** | **10 (8 , 13)** | **1.5 (1.1 , 1.9)** | **-0.1 (-0.3 , 0.1)** |
| **Djibouti** | **6 (5 , 8)** | **1.5 (1.1 , 1.9)** | **16 (12 , 21)** | **1.5 (1.1 , 1.9)** | **0.1 (-0.2 , 0.3)** |
| **Eritrea** | **38 (28 , 50)** | **1.5 (1.1 , 1.9)** | **88 (66 , 115)** | **1.5 (1.1 , 1.9)** | **0.1 (0 , 0.3)** |
| **Ethiopia** | **735 (550 , 953)** | **1.6 (1.2 , 2.1)** | **1542 (1153 , 2001)** | **1.6 (1.2 , 2.1)** | **0.2 (0 , 0.4)** |
| **Kenya** | **372 (277 , 491)** | **1.8 (1.4 , 2.3)** | **827 (626 , 1066)** | **1.8 (1.4 , 2.3)** | **-0.3 (-0.5 , -0.1)** |
| **Madagascar** | **155 (116 , 202)** | **1.5 (1.1 , 1.9)** | **352 (264 , 457)** | **1.5 (1.1 , 1.9)** | **-0.3 (-0.5 , -0.1)** |
| **Malawi** | **123 (92 , 161)** | **1.5 (1.1 , 1.9)** | **239 (178 , 314)** | **1.5 (1.1 , 1.9)** | **-0.3 (-0.5 , -0.2)** |
| **Mozambique** | **173 (129 , 226)** | **1.5 (1.1 , 1.9)** | **381 (282 , 497)** | **1.5 (1.1 , 1.9)** | **-0.2 (-0.4 , 0)** |
| **Rwanda** | **92 (68 , 121)** | **1.5 (1.1 , 1.9)** | **169 (127 , 219)** | **1.5 (1.1 , 1.9)** | **-0.2 (-0.4 , -0.1)** |
| **Somalia** | **93 (68 , 121)** | **1.5 (1.1 , 1.9)** | **260 (192 , 340)** | **1.5 (1.1 , 1.9)** | **-0.2 (-0.6 , 0)** |
| **South Sudan** | **76 (57 , 99)** | **1.5 (1.1 , 1.9)** | **122 (92 , 161)** | **1.5 (1.1 , 1.9)** | **-0.1 (-0.7 , 0.3)** |
| **United Republic of Tanzania** | **262 (192 , 346)** | **1.1 (0.9 , 1.5)** | **654 (487 , 856)** | **1.3 (1 , 1.7)** | **16.8 (6.1 , 29.9)** |
| **Uganda** | **221 (163 , 288)** | **1.5 (1.1 , 1.9)** | **525 (388 , 689)** | **1.5 (1.1 , 1.9)** | **-0.4 (-0.7 , -0.1)** |
| **Zambia** | **101 (75 , 133)** | **1.5 (1.1 , 1.9)** | **236 (176 , 307)** | **1.5 (1.1 , 1.9)** | **-0.4 (-0.8 , -0.1)** |
| **Central Sub-Saharan Africa** | **1183 (901 , 1513)** | **2.4 (1.9 , 3)** | **2836 (2158 , 3624)** | **2.4 (1.9 , 3)** | **-0.2 (-0.5 , 0)** |
| **Angola** | **220 (167 , 282)** | **2.4 (1.9 , 3)** | **644 (488 , 829)** | **2.4 (1.9 , 3)** | **-0.7 (-1.1 , -0.3)** |
| **Central African Republic** | **59 (45 , 75)** | **2.4 (1.9 , 3)** | **114 (87 , 146)** | **2.4 (1.9 , 3)** | **-0.1 (-0.4 , 0.1)** |
| **Congo** | **52 (40 , 67)** | **2.4 (1.9 , 3)** | **117 (90 , 150)** | **2.4 (1.9 , 3)** | **0.5 (0.2 , 0.8)** |
| **Democratic Republic of the Congo** | **821 (626 , 1049)** | **2.4 (1.9 , 3)** | **1891 (1437 , 2411)** | **2.4 (1.9 , 3)** | **-0.2 (-0.6 , 0.1)** |
| **Equatorial Guinea** | **9 (7 , 12)** | **2.4 (1.9 , 3)** | **30 (23 , 39)** | **2.4 (1.9 , 3)** | **0.5 (0 , 1.1)** |
| **Gabon** | **22 (17 , 28)** | **2.4 (1.9 , 3)** | **40 (30 , 50)** | **2.4 (1.9 , 3)** | **0 (-0.3 , 0.3)** |
