# Supplementary material for: Global, regional, and national burden of Guillain–Barré syndrome and its underlying causes from 1990 to 2019
Source: J Neuroinflammation. 2021 Nov 11;18:264. doi: 10.1186/s12974-021-02319-4 (PMC8581128; doi:10.1186/s12974-021-02319-4)
Supplement: Supplementary file 3 — Additional file 3: Table S3. Years lived with disability (YLDs) due to Guillain–Barre syndrome in 1990 and 2019 and the percentage change in the age-standardised rates (ASRs) per 100,000, by location (Generated from data available from http://ghdx.healthdata.org/gbd-results-tool). [file 12974_2021_2319_MOESM3_ESM.doc]

| **Table S3: Years lived with disability (YLDs) due to Guillain-Barre syndrome in 1990 and 2019 and the percentage change in the age-standardised rates (ASRs) per 100,000, by location**  **(Generated from data available from http://ghdx.healthdata.org/gbd-results-tool)** | | | | | |
| --- | --- | --- | --- | --- | --- |
|  | **1990** | | **2019** | | **Percentage change in ASRs per 100,000** |
|  | **No (95% UI)** | **ASRs per 100,000 (95% UI)** | **No (95% UI)** | **ASRs per 100,000 (95% UI)** |
| **Global** | **26696 (16714 , 39628)** | **0.5 (0.3 , 0.8)** | **44407 (28016 , 64777)** | **0.6 (0.4 , 0.8)** | **6.5 (3.6 , 9.5)** |
| **High-income North America** | **2693 (1662 , 3895)** | **0.9 (0.6 , 1.3)** | **6166 (3847 , 8752)** | **1.3 (0.8 , 1.8)** | **41.3 (28.8 , 56.1)** |
| **Canada** | **147 (91 , 217)** | **0.5 (0.3 , 0.7)** | **377 (228 , 566)** | **0.7 (0.4 , 1.1)** | **50.2 (34.5 , 69.1)** |
| **Greenland** | **0 (0 , 1)** | **0.8 (0.5 , 1.3)** | **1 (0 , 1)** | **0.9 (0.5 , 1.3)** | **1 (0.5 , 1.5)** |
| **United States of America** | **2546 (1580 , 3681)** | **0.9 (0.6 , 1.4)** | **5788 (3627 , 8221)** | **1.3 (0.8 , 1.9)** | **41.3 (27.4 , 57.3)** |
| **Australasia** | **89 (55 , 133)** | **0.4 (0.3 , 0.6)** | **166 (100 , 245)** | **0.5 (0.3 , 0.7)** | **13.8 (5 , 24.1)** |
| **Australia** | **64 (39 , 97)** | **0.4 (0.2 , 0.6)** | **133 (80 , 201)** | **0.5 (0.3 , 0.7)** | **24.2 (10.7 , 41.5)** |
| **New Zealand** | **24 (15 , 36)** | **0.7 (0.4 , 1)** | **33 (20 , 48)** | **0.6 (0.4 , 0.9)** | **-12 (-21.9 , -0.6)** |
| **High-income Asia Pacific** | **3002 (1889 , 4448)** | **1.7 (1.1 , 2.6)** | **3465 (2187 , 5008)** | **1.9 (1.2 , 2.8)** | **9.3 (4 , 14.8)** |
| **Brunei Darussalam** | **5 (3 , 7)** | **1.9 (1.2 , 2.8)** | **8 (5 , 12)** | **1.9 (1.2 , 2.7)** | **-0.4 (-0.6 , -0.2)** |
| **Japan** | **2102 (1319 , 3112)** | **1.7 (1.1 , 2.5)** | **2346 (1472 , 3364)** | **1.9 (1.2 , 2.8)** | **12.8 (4.3 , 21.6)** |
| **Singapore** | **57 (36 , 86)** | **1.9 (1.2 , 2.7)** | **106 (67 , 159)** | **1.9 (1.2 , 2.7)** | **0.3 (0.1 , 0.6)** |
| **Republic of Korea** | **838 (527 , 1240)** | **1.9 (1.2 , 2.7)** | **1004 (630 , 1474)** | **1.9 (1.2 , 2.7)** | **0.4 (0.2 , 0.7)** |
| **Western Europe** | **2149 (1308 , 3171)** | **0.5 (0.3 , 0.7)** | **3311 (1985 , 4876)** | **0.6 (0.4 , 0.8)** | **17 (14.1 , 20.6)** |
| **Andorra** | **0 (0 , 0)** | **0.5 (0.3 , 0.8)** | **1 (0 , 1)** | **0.5 (0.3 , 0.8)** | **-0.4 (-0.7 , -0.2)** |
| **Austria** | **55 (35 , 79)** | **0.6 (0.4 , 0.8)** | **113 (68 , 165)** | **0.9 (0.6 , 1.3)** | **53.5 (32.4 , 83)** |
| **Belgium** | **63 (38 , 93)** | **0.5 (0.3 , 0.8)** | **79 (47 , 117)** | **0.5 (0.3 , 0.8)** | **0.3 (0.1 , 0.6)** |
| **Cyprus** | **4 (3 , 6)** | **0.5 (0.3 , 0.8)** | **8 (5 , 12)** | **0.5 (0.3 , 0.8)** | **-0.2 (-0.4 , 0.1)** |
| **Denmark** | **28 (17 , 42)** | **0.5 (0.3 , 0.7)** | **35 (21 , 53)** | **0.5 (0.3 , 0.7)** | **2.1 (-4.2 , 9.8)** |
| **Finland** | **22 (13 , 33)** | **0.4 (0.2 , 0.6)** | **35 (21 , 53)** | **0.5 (0.3 , 0.7)** | **23.4 (14 , 35.4)** |
| **France** | **366 (223 , 540)** | **0.6 (0.3 , 0.8)** | **475 (286 , 710)** | **0.6 (0.3 , 0.8)** | **0.2 (0 , 0.4)** |
| **Germany** | **570 (338 , 843)** | **0.6 (0.4 , 0.9)** | **690 (408 , 1026)** | **0.6 (0.4 , 0.9)** | **0.8 (0.4 , 1.3)** |
| **Greece** | **46 (28 , 70)** | **0.4 (0.2 , 0.6)** | **67 (40 , 100)** | **0.5 (0.3 , 0.7)** | **22.6 (11.9 , 38.2)** |
| **Iceland** | **1 (1 , 2)** | **0.5 (0.3 , 0.8)** | **2 (1 , 3)** | **0.5 (0.3 , 0.8)** | **0.3 (0.2 , 0.5)** |
| **Ireland** | **20 (12 , 30)** | **0.5 (0.3 , 0.8)** | **31 (19 , 46)** | **0.5 (0.3 , 0.8)** | **0.1 (-0.1 , 0.3)** |
| **Israel** | **26 (16 , 38)** | **0.5 (0.3 , 0.8)** | **53 (32 , 78)** | **0.5 (0.3 , 0.8)** | **0.3 (0.2 , 0.5)** |
| **Italy** | **363 (225 , 530)** | **0.6 (0.4 , 0.9)** | **623 (383 , 894)** | **0.7 (0.4 , 1)** | **17 (5.6 , 32.1)** |
| **Luxembourg** | **2 (1 , 4)** | **0.5 (0.3 , 0.8)** | **4 (2 , 6)** | **0.5 (0.3 , 0.8)** | **0.7 (0.4 , 1.3)** |
| **Malta** | **2 (1 , 3)** | **0.5 (0.3 , 0.8)** | **3 (2 , 5)** | **0.5 (0.3 , 0.8)** | **0.7 (0.5 , 1)** |
| **Monaco** | **0 (0 , 0)** | **0.5 (0.3 , 0.8)** | **0 (0 , 0)** | **0.5 (0.3 , 0.8)** | **0.3 (0.1 , 0.6)** |
| **Netherlands** | **71 (43 , 106)** | **0.4 (0.3 , 0.6)** | **119 (70 , 178)** | **0.5 (0.3 , 0.8)** | **23.7 (13.5 , 38.7)** |
| **Norway** | **41 (25 , 59)** | **0.8 (0.5 , 1.2)** | **54 (33 , 80)** | **0.8 (0.5 , 1.2)** | **0.6 (0.3 , 0.9)** |
| **Portugal** | **66 (40 , 98)** | **0.6 (0.4 , 0.8)** | **87 (53 , 130)** | **0.6 (0.4 , 0.9)** | **7.3 (1.2 , 14.1)** |
| **San Marino** | **0 (0 , 0)** | **0.5 (0.3 , 0.8)** | **0 (0 , 0)** | **0.5 (0.3 , 0.8)** | **-0.5 (-1 , 0)** |
| **Spain** | **173 (105 , 262)** | **0.4 (0.2 , 0.6)** | **288 (172 , 434)** | **0.5 (0.3 , 0.7)** | **17.9 (9.1 , 29.8)** |
| **Sweden** | **34 (20 , 51)** | **0.3 (0.2 , 0.5)** | **89 (53 , 134)** | **0.7 (0.4 , 1)** | **102.6 (85.8 , 122.4)** |
| **Switzerland** | **43 (26 , 64)** | **0.5 (0.3 , 0.8)** | **61 (36 , 91)** | **0.5 (0.3 , 0.8)** | **0.5 (0.3 , 0.8)** |
| **United Kingdom** | **152 (90 , 232)** | **0.2 (0.1 , 0.3)** | **391 (233 , 589)** | **0.5 (0.3 , 0.7)** | **104.7 (90.1 , 124.4)** |
| **Southern Latin America** | **359 (219 , 527)** | **0.7 (0.4 , 1.1)** | **522 (317 , 767)** | **0.7 (0.4 , 1.1)** | **0.1 (0.1 , 0.2)** |
| **Argentina** | **244 (149 , 359)** | **0.7 (0.4 , 1.1)** | **347 (210 , 510)** | **0.7 (0.4 , 1.1)** | **0.1 (0 , 0.2)** |
| **Chile** | **90 (55 , 132)** | **0.7 (0.4 , 1.1)** | **146 (88 , 214)** | **0.7 (0.4 , 1.1)** | **0.2 (0.1 , 0.2)** |
| **Uruguay** | **25 (15 , 37)** | **0.7 (0.4 , 1.1)** | **29 (17 , 42)** | **0.7 (0.4 , 1.1)** | **-0.1 (-0.1 , 0)** |
| **Eastern Europe** | **1307 (804 , 1955)** | **0.6 (0.3 , 0.8)** | **1279 (791 , 1896)** | **0.6 (0.3 , 0.8)** | **0.4 (0.2 , 0.6)** |
| **Belarus** | **57 (35 , 84)** | **0.5 (0.3 , 0.8)** | **55 (33 , 81)** | **0.5 (0.3 , 0.8)** | **0.1 (0 , 0.3)** |
| **Estonia** | **9 (5 , 13)** | **0.5 (0.3 , 0.8)** | **8 (5 , 11)** | **0.5 (0.3 , 0.8)** | **0.2 (0 , 0.5)** |
| **Latvia** | **15 (9 , 22)** | **0.5 (0.3 , 0.8)** | **11 (7 , 17)** | **0.5 (0.3 , 0.8)** | **0.2 (0 , 0.4)** |
| **Lithuania** | **20 (12 , 29)** | **0.5 (0.3 , 0.8)** | **17 (10 , 25)** | **0.5 (0.3 , 0.8)** | **0 (-0.1 , 0.2)** |
| **Republic of Moldova** | **23 (14 , 35)** | **0.5 (0.3 , 0.8)** | **21 (13 , 31)** | **0.5 (0.3 , 0.8)** | **0.1 (0 , 0.2)** |
| **Russian Federation** | **873 (541 , 1305)** | **0.6 (0.3 , 0.8)** | **896 (555 , 1333)** | **0.6 (0.3 , 0.9)** | **0.3 (0.1 , 0.6)** |
| **Ukraine** | **311 (192 , 463)** | **0.6 (0.3 , 0.9)** | **272 (167 , 404)** | **0.6 (0.3 , 0.9)** | **0.2 (0.1 , 0.4)** |
| **Central Europe** | **543 (332 , 810)** | **0.4 (0.3 , 0.6)** | **550 (331 , 807)** | **0.4 (0.3 , 0.6)** | **-1.9 (-5.6 , 2.7)** |
| **Albania** | **13 (8 , 20)** | **0.4 (0.3 , 0.6)** | **13 (7 , 19)** | **0.4 (0.3 , 0.6)** | **0 (-0.3 , 0.3)** |
| **Bosnia and Herzegovina** | **18 (11 , 27)** | **0.4 (0.3 , 0.6)** | **15 (9 , 23)** | **0.4 (0.3 , 0.6)** | **0.2 (0 , 0.4)** |
| **Bulgaria** | **40 (24 , 59)** | **0.4 (0.3 , 0.6)** | **34 (20 , 52)** | **0.4 (0.3 , 0.6)** | **-0.1 (-0.3 , 0)** |
| **Croatia** | **22 (13 , 32)** | **0.4 (0.3 , 0.6)** | **21 (12 , 31)** | **0.4 (0.3 , 0.6)** | **0.4 (0.1 , 0.7)** |
| **Czechia** | **46 (27 , 67)** | **0.4 (0.3 , 0.6)** | **52 (31 , 77)** | **0.4 (0.3 , 0.6)** | **0.4 (0.2 , 0.7)** |
| **Hungary** | **47 (28 , 69)** | **0.4 (0.3 , 0.6)** | **47 (28 , 70)** | **0.4 (0.3 , 0.6)** | **0.1 (0 , 0.2)** |
| **Montenegro** | **3 (2 , 4)** | **0.4 (0.3 , 0.6)** | **3 (2 , 4)** | **0.4 (0.3 , 0.6)** | **0.2 (0.1 , 0.4)** |
| **North Macedonia** | **8 (5 , 13)** | **0.4 (0.3 , 0.6)** | **10 (6 , 15)** | **0.4 (0.3 , 0.6)** | **0 (-0.1 , 0.2)** |
| **Poland** | **178 (109 , 272)** | **0.5 (0.3 , 0.7)** | **190 (117 , 276)** | **0.4 (0.3 , 0.6)** | **-5.7 (-15.9 , 7.6)** |
| **Romania** | **102 (61 , 151)** | **0.4 (0.3 , 0.6)** | **93 (56 , 138)** | **0.4 (0.3 , 0.6)** | **0 (-0.1 , 0.1)** |
| **Serbia** | **36 (22 , 54)** | **0.4 (0.2 , 0.5)** | **37 (22 , 56)** | **0.4 (0.2 , 0.6)** | **0.4 (0.2 , 0.7)** |
| **Slovakia** | **23 (14 , 34)** | **0.4 (0.3 , 0.6)** | **25 (15 , 38)** | **0.4 (0.3 , 0.6)** | **0.1 (0 , 0.3)** |
| **Slovenia** | **9 (5 , 13)** | **0.4 (0.3 , 0.6)** | **10 (6 , 15)** | **0.4 (0.3 , 0.6)** | **0.6 (0.2 , 1.1)** |
| **Central Asia** | **356 (218 , 528)** | **0.6 (0.3 , 0.8)** | **501 (313 , 751)** | **0.6 (0.3 , 0.8)** | **0 (-0.7 , 0.6)** |
| **Armenia** | **18 (11 , 27)** | **0.6 (0.3 , 0.8)** | **18 (11 , 27)** | **0.6 (0.3 , 0.8)** | **0.3 (0.2 , 0.5)** |
| **Azerbaijan** | **38 (23 , 56)** | **0.6 (0.3 , 0.8)** | **57 (35 , 86)** | **0.6 (0.3 , 0.8)** | **0.4 (0.1 , 0.7)** |
| **Georgia** | **32 (19 , 47)** | **0.6 (0.3 , 0.8)** | **22 (13 , 32)** | **0.5 (0.3 , 0.8)** | **-7.3 (-17.5 , 5.2)** |
| **Kazakhstan** | **86 (54 , 129)** | **0.6 (0.3 , 0.8)** | **102 (64 , 153)** | **0.6 (0.3 , 0.8)** | **0.1 (-0.1 , 0.3)** |
| **Kyrgyzstan** | **23 (14 , 34)** | **0.6 (0.3 , 0.8)** | **34 (21 , 51)** | **0.6 (0.3 , 0.8)** | **0.3 (0.1 , 0.6)** |
| **Mongolia** | **11 (7 , 16)** | **0.6 (0.3 , 0.8)** | **18 (11 , 27)** | **0.6 (0.3 , 0.8)** | **0.3 (0.1 , 0.5)** |
| **Tajikistan** | **27 (16 , 40)** | **0.6 (0.3 , 0.8)** | **48 (30 , 72)** | **0.6 (0.3 , 0.8)** | **0.4 (0.1 , 0.7)** |
| **Turkmenistan** | **18 (11 , 27)** | **0.6 (0.3 , 0.8)** | **27 (17 , 41)** | **0.6 (0.3 , 0.8)** | **0.2 (-0.1 , 0.5)** |
| **Uzbekistan** | **104 (64 , 156)** | **0.6 (0.3 , 0.8)** | **175 (109 , 262)** | **0.6 (0.3 , 0.8)** | **0.3 (0.1 , 0.5)** |
| **Central Latin America** | **1674 (1038 , 2530)** | **1.1 (0.7 , 1.7)** | **2811 (1742 , 4128)** | **1.2 (0.7 , 1.7)** | **2.7 (-0.9 , 7.1)** |
| **Colombia** | **308 (190 , 460)** | **1.1 (0.6 , 1.5)** | **500 (305 , 731)** | **1 (0.6 , 1.5)** | **-0.2 (-0.4 , 0)** |
| **Costa Rica** | **29 (18 , 44)** | **1.1 (0.6 , 1.5)** | **49 (30 , 72)** | **1 (0.6 , 1.5)** | **-0.3 (-0.6 , -0.1)** |
| **El Salvador** | **46 (28 , 69)** | **1 (0.6 , 1.4)** | **60 (37 , 87)** | **1 (0.6 , 1.4)** | **2.3 (-4 , 9.7)** |
| **Guatemala** | **78 (48 , 118)** | **1.1 (0.6 , 1.5)** | **169 (104 , 252)** | **1 (0.6 , 1.5)** | **-0.7 (-1.1 , -0.1)** |
| **Honduras** | **40 (25 , 62)** | **0.9 (0.6 , 1.4)** | **87 (53 , 129)** | **1 (0.6 , 1.4)** | **2.8 (-3.7 , 10.7)** |
| **Mexico** | **933 (571 , 1412)** | **1.2 (0.7 , 1.8)** | **1544 (963 , 2274)** | **1.3 (0.8 , 1.8)** | **5.3 (-1.3 , 14.1)** |
| **Nicaragua** | **37 (23 , 57)** | **1 (0.6 , 1.5)** | **63 (39 , 93)** | **1 (0.6 , 1.5)** | **0.5 (0.1 , 1)** |
| **Panama** | **23 (14 , 35)** | **1.1 (0.6 , 1.5)** | **45 (27 , 65)** | **1.1 (0.7 , 1.6)** | **2.3 (0.7 , 5.9)** |
| **Venezuela (Bolivarian Republic of)** | **178 (111 , 267)** | **1 (0.6 , 1.5)** | **295 (178 , 433)** | **1 (0.6 , 1.5)** | **-0.2 (-0.3 , -0.1)** |
| **Andean Latin America** | **222 (139 , 326)** | **0.7 (0.4 , 1)** | **406 (255 , 592)** | **0.7 (0.4 , 1)** | **-6.8 (-8.6 , -4.7)** |
| **Bolivia (Plurinational State of)** | **36 (23 , 54)** | **0.7 (0.4 , 1)** | **77 (48 , 113)** | **0.7 (0.4 , 1)** | **1 (0.6 , 2.2)** |
| **Ecuador** | **59 (37 , 85)** | **0.7 (0.5 , 1.1)** | **90 (57 , 131)** | **0.5 (0.3 , 0.8)** | **-26.1 (-32 , -19)** |
| **Peru** | **127 (79 , 188)** | **0.7 (0.4 , 1)** | **239 (148 , 351)** | **0.7 (0.4 , 1)** | **0.6 (0.1 , 1.9)** |
| **Caribbean** | **199 (124 , 298)** | **0.6 (0.4 , 0.9)** | **290 (180 , 427)** | **0.6 (0.4 , 0.9)** | **0 (-0.1 , 0.1)** |
| **Antigua and Barbuda** | **0 (0 , 1)** | **0.6 (0.4 , 0.9)** | **1 (0 , 1)** | **0.6 (0.4 , 0.9)** | **0.5 (0.3 , 0.8)** |
| **Barbados** | **2 (1 , 2)** | **0.6 (0.4 , 0.9)** | **2 (1 , 3)** | **0.6 (0.4 , 0.9)** | **0.6 (0.3 , 1.1)** |
| **Belize** | **1 (1 , 2)** | **0.6 (0.4 , 0.9)** | **2 (1 , 3)** | **0.6 (0.4 , 0.9)** | **1.8 (0.6 , 4.6)** |
| **Bermuda** | **0 (0 , 1)** | **0.6 (0.4 , 0.9)** | **0 (0 , 1)** | **0.6 (0.4 , 0.9)** | **0.3 (0.1 , 0.4)** |
| **Bahamas** | **1 (1 , 2)** | **0.6 (0.4 , 0.9)** | **2 (1 , 3)** | **0.6 (0.4 , 0.9)** | **0.2 (0.1 , 0.4)** |
| **Cuba** | **64 (40 , 94)** | **0.6 (0.4 , 0.9)** | **79 (47 , 118)** | **0.6 (0.4 , 0.9)** | **0 (-0.2 , 0.2)** |
| **Dominica** | **0 (0 , 1)** | **0.6 (0.4 , 0.9)** | **0 (0 , 1)** | **0.6 (0.4 , 0.9)** | **0.9 (0.3 , 1.5)** |
| **Dominican Republic** | **39 (24 , 58)** | **0.6 (0.4 , 0.9)** | **63 (40 , 94)** | **0.6 (0.4 , 0.9)** | **0.2 (0 , 0.4)** |
| **Grenada** | **0 (0 , 1)** | **0.6 (0.4 , 0.9)** | **1 (0 , 1)** | **0.6 (0.4 , 0.9)** | **0.5 (0.2 , 1)** |
| **Guyana** | **4 (3 , 6)** | **0.6 (0.4 , 0.9)** | **4 (3 , 7)** | **0.6 (0.4 , 0.9)** | **-0.2 (-0.3 , -0.1)** |
| **Haiti** | **34 (21 , 52)** | **0.6 (0.4 , 0.9)** | **67 (42 , 101)** | **0.6 (0.4 , 0.9)** | **0 (-0.2 , 0.3)** |
| **Jamaica** | **13 (8 , 20)** | **0.6 (0.4 , 0.9)** | **17 (11 , 25)** | **0.6 (0.4 , 0.9)** | **0.2 (0.1 , 0.4)** |
| **Puerto Rico** | **22 (14 , 32)** | **0.6 (0.4 , 0.9)** | **25 (15 , 38)** | **0.6 (0.4 , 0.9)** | **-0.1 (-0.2 , 0)** |
| **Saint Kitts and Nevis** | **0 (0 , 0)** | **0.6 (0.4 , 0.9)** | **0 (0 , 1)** | **0.6 (0.4 , 0.9)** | **0.4 (0.2 , 0.7)** |
| **Saint Lucia** | **1 (0 , 1)** | **0.6 (0.4 , 0.9)** | **1 (1 , 2)** | **0.6 (0.4 , 0.9)** | **0.6 (0.4 , 0.9)** |
| **Saint Vincent and the Grenadines** | **1 (0 , 1)** | **0.6 (0.4 , 0.9)** | **1 (0 , 1)** | **0.6 (0.4 , 0.9)** | **0.9 (0.5 , 1.4)** |
| **Suriname** | **2 (1 , 3)** | **0.6 (0.4 , 0.9)** | **4 (2 , 5)** | **0.6 (0.4 , 0.9)** | **-0.3 (-0.5 , -0.1)** |
| **Trinidad and Tobago** | **7 (4 , 10)** | **0.6 (0.4 , 0.9)** | **9 (5 , 13)** | **0.6 (0.4 , 0.9)** | **0.2 (0.1 , 0.3)** |
| **United States Virgin Islands** | **1 (0 , 1)** | **0.6 (0.4 , 0.9)** | **1 (0 , 1)** | **0.6 (0.4 , 0.9)** | **0 (-0.1 , 0.2)** |
| **Tropical Latin America** | **844 (518 , 1283)** | **0.7 (0.4 , 1)** | **948 (581 , 1399)** | **0.4 (0.2 , 0.6)** | **-40.3 (-49.9 , -31.3)** |
| **Brazil** | **834 (512 , 1268)** | **0.7 (0.4 , 1)** | **927 (567 , 1368)** | **0.4 (0.2 , 0.6)** | **-40.7 (-50.4 , -31.6)** |
| **Paraguay** | **11 (6 , 16)** | **0.3 (0.2 , 0.5)** | **22 (13 , 33)** | **0.3 (0.2 , 0.5)** | **0.2 (-5.9 , 7.5)** |
| **East Asia** | **2414 (1466 , 3681)** | **0.2 (0.1 , 0.3)** | **3515 (2120 , 5320)** | **0.2 (0.1 , 0.4)** | **11.4 (4.7 , 18.7)** |
| **China** | **2307 (1404 , 3517)** | **0.2 (0.1 , 0.3)** | **3346 (2019 , 5069)** | **0.2 (0.1 , 0.4)** | **11.3 (4.3 , 18.9)** |
| **Democratic People's Republic of Korea** | **50 (31 , 76)** | **0.3 (0.2 , 0.4)** | **67 (41 , 101)** | **0.3 (0.2 , 0.4)** | **0.3 (-0.1 , 0.8)** |
| **Taiwan (Province of China)** | **57 (35 , 86)** | **0.3 (0.2 , 0.4)** | **103 (60 , 156)** | **0.4 (0.2 , 0.5)** | **24.2 (11.2 , 51)** |
| **Southeast Asia** | **1417 (861 , 2158)** | **0.3 (0.2 , 0.5)** | **2096 (1299 , 3172)** | **0.3 (0.2 , 0.5)** | **0.3 (0.1 , 0.6)** |
| **Cambodia** | **30 (18 , 46)** | **0.3 (0.2 , 0.5)** | **48 (30 , 73)** | **0.3 (0.2 , 0.5)** | **0.1 (-0.2 , 0.4)** |
| **Indonesia** | **589 (357 , 897)** | **0.3 (0.2 , 0.5)** | **830 (509 , 1249)** | **0.3 (0.2 , 0.5)** | **0 (-0.1 , 0.1)** |
| **Lao People's Democratic Republic** | **12 (7 , 18)** | **0.3 (0.2 , 0.5)** | **20 (13 , 31)** | **0.3 (0.2 , 0.5)** | **0.2 (0.1 , 0.4)** |
| **Malaysia** | **50 (31 , 77)** | **0.3 (0.2 , 0.5)** | **92 (57 , 141)** | **0.3 (0.2 , 0.5)** | **0.1 (0 , 0.3)** |
| **Maldives** | **1 (0 , 1)** | **0.3 (0.2 , 0.5)** | **1 (1 , 2)** | **0.3 (0.2 , 0.5)** | **0 (-0.7 , 0.8)** |
| **Mauritius** | **3 (2 , 5)** | **0.3 (0.2 , 0.5)** | **4 (2 , 6)** | **0.3 (0.2 , 0.5)** | **0.1 (0 , 0.2)** |
| **Myanmar** | **118 (72 , 181)** | **0.3 (0.2 , 0.5)** | **160 (100 , 244)** | **0.3 (0.2 , 0.5)** | **-0.1 (-0.3 , 0)** |
| **Philippines** | **202 (122 , 305)** | **0.3 (0.2 , 0.5)** | **359 (222 , 543)** | **0.3 (0.2 , 0.5)** | **-0.2 (-0.4 , 0)** |
| **Sri Lanka** | **49 (30 , 75)** | **0.3 (0.2 , 0.5)** | **67 (41 , 102)** | **0.3 (0.2 , 0.5)** | **-0.4 (-0.8 , -0.1)** |
| **Seychelles** | **0 (0 , 0)** | **0.3 (0.2 , 0.5)** | **0 (0 , 0)** | **0.3 (0.2 , 0.5)** | **0.3 (0 , 0.5)** |
| **Thailand** | **162 (100 , 247)** | **0.3 (0.2 , 0.5)** | **223 (137 , 336)** | **0.3 (0.2 , 0.5)** | **0.1 (0 , 0.1)** |
| **Timor-Leste** | **2 (1 , 3)** | **0.3 (0.2 , 0.5)** | **4 (2 , 6)** | **0.3 (0.2 , 0.5)** | **0 (-0.1 , 0.2)** |
| **Viet Nam** | **197 (120 , 303)** | **0.3 (0.2 , 0.5)** | **284 (176 , 431)** | **0.3 (0.2 , 0.5)** | **0.2 (0 , 0.4)** |
| **Oceania** | **18 (11 , 28)** | **0.3 (0.2 , 0.5)** | **38 (23 , 58)** | **0.3 (0.2 , 0.5)** | **0 (-0.1 , 0)** |
| **American Samoa** | **0 (0 , 0)** | **0.3 (0.2 , 0.5)** | **0 (0 , 0)** | **0.3 (0.2 , 0.5)** | **-0.1 (-0.3 , 0)** |
| **Cook Islands** | **0 (0 , 0)** | **0.3 (0.2 , 0.5)** | **0 (0 , 0)** | **0.3 (0.2 , 0.5)** | **-0.2 (-0.6 , 0.1)** |
| **Micronesia (Federated States of)** | **0 (0 , 0)** | **0.3 (0.2 , 0.5)** | **0 (0 , 0)** | **0.3 (0.2 , 0.5)** | **-0.1 (-0.3 , 0.1)** |
| **Fiji** | **2 (1 , 3)** | **0.3 (0.2 , 0.5)** | **3 (2 , 4)** | **0.3 (0.2 , 0.5)** | **-0.2 (-0.4 , 0)** |
| **Guam** | **0 (0 , 1)** | **0.3 (0.2 , 0.5)** | **1 (0 , 1)** | **0.3 (0.2 , 0.5)** | **-0.2 (-0.3 , 0)** |
| **Kiribati** | **0 (0 , 0)** | **0.3 (0.2 , 0.5)** | **0 (0 , 1)** | **0.3 (0.2 , 0.5)** | **-0.1 (-0.2 , 0)** |
| **Marshall Islands** | **0 (0 , 0)** | **0.3 (0.2 , 0.5)** | **0 (0 , 0)** | **0.3 (0.2 , 0.5)** | **0.4 (0.1 , 0.8)** |
| **Nauru** | **0 (0 , 0)** | **0.3 (0.2 , 0.5)** | **0 (0 , 0)** | **0.3 (0.2 , 0.5)** | **-0.1 (-0.6 , 0.3)** |
| **Niue** | **0 (0 , 0)** | **0.3 (0.2 , 0.5)** | **0 (0 , 0)** | **0.3 (0.2 , 0.5)** | **0.1 (0 , 0.3)** |
| **Northern Mariana Islands** | **0 (0 , 0)** | **0.3 (0.2 , 0.5)** | **0 (0 , 0)** | **0.3 (0.2 , 0.5)** | **-0.3 (-0.8 , 0.3)** |
| **Palau** | **0 (0 , 0)** | **0.3 (0.2 , 0.5)** | **0 (0 , 0)** | **0.3 (0.2 , 0.5)** | **0.3 (-0.1 , 0.6)** |
| **Papua New Guinea** | **12 (7 , 18)** | **0.3 (0.2 , 0.5)** | **28 (17 , 43)** | **0.3 (0.2 , 0.5)** | **0 (-0.1 , 0.1)** |
| **Samoa** | **0 (0 , 1)** | **0.3 (0.2 , 0.5)** | **1 (0 , 1)** | **0.3 (0.2 , 0.5)** | **0.1 (-0.1 , 0.3)** |
| **Solomon Islands** | **1 (1 , 1)** | **0.3 (0.2 , 0.5)** | **2 (1 , 3)** | **0.3 (0.2 , 0.5)** | **-0.2 (-0.5 , 0.1)** |
| **Tokelau** | **0 (0 , 0)** | **0.3 (0.2 , 0.5)** | **0 (0 , 0)** | **0.3 (0.2 , 0.5)** | **0.3 (-0.1 , 0.7)** |
| **Tonga** | **0 (0 , 0)** | **0.3 (0.2 , 0.5)** | **0 (0 , 0)** | **0.3 (0.2 , 0.5)** | **-0.1 (-0.3 , 0)** |
| **Tuvalu** | **0 (0 , 0)** | **0.3 (0.2 , 0.5)** | **0 (0 , 0)** | **0.3 (0.2 , 0.5)** | **0.6 (0.1 , 1.2)** |
| **Vanuatu** | **0 (0 , 1)** | **0.3 (0.2 , 0.5)** | **1 (1 , 1)** | **0.3 (0.2 , 0.5)** | **-0.2 (-0.3 , 0)** |
| **North Africa and Middle East** | **1563 (973 , 2359)** | **0.5 (0.3 , 0.8)** | **3073 (1903 , 4538)** | **0.5 (0.3 , 0.8)** | **0.3 (-0.9 , 1.5)** |
| **Afghanistan** | **53 (33 , 80)** | **0.5 (0.3 , 0.8)** | **164 (102 , 247)** | **0.5 (0.3 , 0.8)** | **-0.3 (-0.8 , 0.2)** |
| **Algeria** | **113 (71 , 171)** | **0.5 (0.3 , 0.8)** | **216 (134 , 320)** | **0.5 (0.3 , 0.8)** | **-0.1 (-0.2 , 0.1)** |
| **Bahrain** | **2 (1 , 3)** | **0.5 (0.3 , 0.8)** | **8 (5 , 12)** | **0.5 (0.3 , 0.8)** | **-0.4 (-0.9 , 0)** |
| **Egypt** | **256 (160 , 386)** | **0.5 (0.3 , 0.8)** | **483 (301 , 720)** | **0.5 (0.3 , 0.8)** | **0 (-0.2 , 0.3)** |
| **Iran (Islamic Republic of)** | **248 (153 , 376)** | **0.5 (0.3 , 0.7)** | **421 (257 , 632)** | **0.5 (0.3 , 0.7)** | **0.2 (-0.1 , 0.4)** |
| **Iraq** | **77 (48 , 115)** | **0.5 (0.3 , 0.8)** | **198 (124 , 298)** | **0.5 (0.3 , 0.8)** | **0 (0 , 0)** |
| **Jordan** | **19 (12 , 28)** | **0.6 (0.4 , 0.9)** | **72 (45 , 108)** | **0.7 (0.4 , 1)** | **11.4 (3.6 , 20.5)** |
| **Kuwait** | **8 (5 , 12)** | **0.5 (0.3 , 0.8)** | **24 (15 , 37)** | **0.5 (0.3 , 0.8)** | **2 (-4 , 9.2)** |
| **Lebanon** | **16 (10 , 24)** | **0.5 (0.3 , 0.8)** | **28 (17 , 41)** | **0.5 (0.3 , 0.8)** | **0.2 (-0.1 , 0.6)** |
| **Libya** | **14 (9 , 21)** | **0.4 (0.2 , 0.6)** | **31 (19 , 47)** | **0.5 (0.3 , 0.7)** | **19.1 (6.4 , 36.4)** |
| **Morocco** | **116 (72 , 175)** | **0.5 (0.3 , 0.8)** | **189 (117 , 278)** | **0.5 (0.3 , 0.8)** | **0 (-0.1 , 0.1)** |
| **Palestine** | **9 (6 , 13)** | **0.5 (0.3 , 0.8)** | **23 (14 , 34)** | **0.5 (0.3 , 0.8)** | **-0.5 (-1 , -0.1)** |
| **Oman** | **8 (5 , 13)** | **0.5 (0.3 , 0.8)** | **21 (13 , 33)** | **0.5 (0.3 , 0.8)** | **-0.2 (-0.5 , 0.1)** |
| **Qatar** | **2 (1 , 3)** | **0.5 (0.3 , 0.8)** | **14 (8 , 21)** | **0.5 (0.3 , 0.8)** | **0.3 (-0.3 , 1)** |
| **Saudi Arabia** | **70 (44 , 106)** | **0.5 (0.3 , 0.8)** | **177 (107 , 268)** | **0.5 (0.3 , 0.8)** | **0.3 (0.1 , 0.4)** |
| **Sudan** | **89 (56 , 134)** | **0.5 (0.3 , 0.8)** | **184 (116 , 278)** | **0.5 (0.3 , 0.8)** | **0 (-0.1 , 0.2)** |
| **Syrian Arab Republic** | **56 (34 , 84)** | **0.5 (0.3 , 0.8)** | **76 (47 , 113)** | **0.5 (0.3 , 0.8)** | **-0.1 (-0.4 , 0.3)** |
| **Tunisia** | **39 (25 , 59)** | **0.5 (0.3 , 0.8)** | **65 (40 , 96)** | **0.5 (0.3 , 0.8)** | **0 (-0.2 , 0.1)** |
| **Turkey** | **300 (186 , 450)** | **0.6 (0.3 , 0.8)** | **487 (299 , 724)** | **0.6 (0.4 , 0.8)** | **0.8 (-5 , 7.6)** |
| **United Arab Emirates** | **8 (5 , 13)** | **0.5 (0.3 , 0.8)** | **49 (28 , 77)** | **0.5 (0.3 , 0.8)** | **-0.1 (-0.6 , 0.4)** |
| **Yemen** | **58 (36 , 88)** | **0.5 (0.3 , 0.8)** | **140 (88 , 212)** | **0.5 (0.3 , 0.8)** | **0.3 (0 , 0.5)** |
| **South Asia** | **4971 (3078 , 7495)** | **0.5 (0.3 , 0.8)** | **8967 (5591 , 13346)** | **0.5 (0.3 , 0.8)** | **1.4 (-2.9 , 5.6)** |
| **Bangladesh** | **404 (248 , 607)** | **0.4 (0.3 , 0.7)** | **677 (418 , 1005)** | **0.4 (0.3 , 0.7)** | **-0.1 (-0.4 , 0.3)** |
| **Bhutan** | **2 (1 , 3)** | **0.5 (0.3 , 0.7)** | **3 (2 , 5)** | **0.4 (0.3 , 0.7)** | **-1.2 (-1.7 , -0.8)** |
| **India** | **4020 (2509 , 6054)** | **0.5 (0.3 , 0.8)** | **7235 (4503 , 10793)** | **0.5 (0.3 , 0.8)** | **2 (-3.5 , 7.3)** |
| **Nepal** | **68 (42 , 102)** | **0.4 (0.3 , 0.6)** | **98 (61 , 145)** | **0.3 (0.2 , 0.5)** | **-15.7 (-22.5 , -7.4)** |
| **Pakistan** | **477 (299 , 713)** | **0.5 (0.3 , 0.7)** | **954 (598 , 1435)** | **0.5 (0.3 , 0.7)** | **0.2 (-0.1 , 0.5)** |
| **Southern Sub-Saharan Africa** | **489 (308 , 725)** | **1 (0.6 , 1.5)** | **765 (478 , 1144)** | **1 (0.6 , 1.5)** | **-0.1 (-0.3 , 0)** |
| **Botswana** | **11 (7 , 17)** | **0.9 (0.6 , 1.4)** | **21 (13 , 31)** | **0.9 (0.6 , 1.4)** | **0.2 (-0.1 , 0.4)** |
| **Lesotho** | **16 (10 , 23)** | **0.9 (0.6 , 1.4)** | **19 (12 , 28)** | **0.9 (0.6 , 1.4)** | **-0.1 (-0.4 , 0.1)** |
| **Namibia** | **12 (8 , 18)** | **0.9 (0.6 , 1.4)** | **21 (13 , 32)** | **0.9 (0.6 , 1.4)** | **-0.4 (-0.6 , -0.2)** |
| **South Africa** | **356 (224 , 534)** | **1 (0.6 , 1.5)** | **564 (353 , 851)** | **1 (0.6 , 1.5)** | **0.1 (-0.1 , 0.2)** |
| **Eswatini** | **7 (4 , 10)** | **0.9 (0.6 , 1.4)** | **10 (6 , 15)** | **0.9 (0.6 , 1.4)** | **-0.1 (-0.5 , 0.3)** |
| **Zimbabwe** | **87 (54 , 131)** | **0.9 (0.6 , 1.4)** | **130 (82 , 193)** | **0.9 (0.6 , 1.4)** | **-0.8 (-1.2 , -0.4)** |
| **Western Sub-Saharan Africa** | **1287 (801 , 1924)** | **0.7 (0.5 , 1.1)** | **3049 (1895 , 4575)** | **0.7 (0.5 , 1.1)** | **-0.4 (-0.9 , 0)** |
| **Benin** | **30 (19 , 45)** | **0.7 (0.4 , 1.1)** | **80 (50 , 120)** | **0.7 (0.4 , 1.1)** | **0 (-0.3 , 0.2)** |
| **Burkina Faso** | **61 (38 , 91)** | **0.7 (0.4 , 1.1)** | **143 (89 , 215)** | **0.7 (0.4 , 1.1)** | **-0.1 (-0.3 , 0.1)** |
| **Cameroon** | **66 (41 , 99)** | **0.7 (0.4 , 1.1)** | **186 (117 , 279)** | **0.7 (0.4 , 1.1)** | **0 (-0.1 , 0.1)** |
| **Cabo Verde** | **2 (1 , 3)** | **0.7 (0.4 , 1.1)** | **4 (2 , 6)** | **0.7 (0.4 , 1.1)** | **0.7 (0.1 , 1.5)** |
| **Chad** | **38 (24 , 57)** | **0.7 (0.4 , 1.1)** | **102 (63 , 153)** | **0.7 (0.4 , 1.1)** | **0.7 (0.3 , 1.1)** |
| **CÃ´te d'Ivoire** | **76 (47 , 115)** | **0.7 (0.4 , 1.1)** | **169 (106 , 251)** | **0.7 (0.4 , 1.1)** | **-0.1 (-0.3 , 0)** |
| **Gambia** | **6 (4 , 9)** | **0.7 (0.4 , 1.1)** | **14 (9 , 22)** | **0.7 (0.4 , 1.1)** | **-0.4 (-0.7 , -0.1)** |
| **Ghana** | **95 (59 , 143)** | **0.7 (0.4 , 1.1)** | **206 (131 , 311)** | **0.7 (0.4 , 1.1)** | **-0.5 (-0.7 , -0.2)** |
| **Guinea** | **40 (25 , 59)** | **0.7 (0.4 , 1.1)** | **80 (50 , 120)** | **0.7 (0.4 , 1.1)** | **0 (-0.1 , 0.1)** |
| **Guinea-Bissau** | **6 (4 , 9)** | **0.7 (0.4 , 1.1)** | **12 (7 , 18)** | **0.7 (0.4 , 1.1)** | **-0.2 (-0.5 , 0)** |
| **Liberia** | **13 (8 , 19)** | **0.7 (0.4 , 1.1)** | **31 (20 , 47)** | **0.7 (0.4 , 1.1)** | **0 (-0.3 , 0.2)** |
| **Mali** | **55 (34 , 83)** | **0.7 (0.4 , 1.1)** | **138 (86 , 207)** | **0.7 (0.4 , 1.1)** | **0.3 (0.2 , 0.5)** |
| **Mauritania** | **13 (8 , 20)** | **0.7 (0.4 , 1.1)** | **26 (17 , 39)** | **0.7 (0.4 , 1.1)** | **0.3 (0.1 , 0.5)** |
| **Niger** | **50 (31 , 75)** | **0.7 (0.4 , 1.1)** | **144 (89 , 217)** | **0.7 (0.4 , 1.1)** | **-0.3 (-0.5 , 0.1)** |
| **Nigeria** | **640 (397 , 959)** | **0.8 (0.5 , 1.2)** | **1509 (935 , 2282)** | **0.8 (0.5 , 1.2)** | **-0.8 (-1.6 , -0.1)** |
| **Sao Tome and Principe** | **1 (0 , 1)** | **0.7 (0.4 , 1.1)** | **1 (1 , 2)** | **0.7 (0.4 , 1.1)** | **0.3 (0.1 , 0.6)** |
| **Senegal** | **48 (30 , 72)** | **0.7 (0.4 , 1.1)** | **98 (62 , 147)** | **0.7 (0.4 , 1.1)** | **-0.1 (-0.3 , 0.1)** |
| **Sierra Leone** | **24 (15 , 35)** | **0.7 (0.4 , 1.1)** | **53 (33 , 80)** | **0.7 (0.4 , 1.1)** | **0 (-0.2 , 0.1)** |
| **Togo** | **23 (14 , 34)** | **0.7 (0.4 , 1.1)** | **51 (33 , 77)** | **0.7 (0.4 , 1.1)** | **-0.4 (-0.8 , -0.2)** |
| **Eastern Sub-Saharan Africa** | **748 (456 , 1132)** | **0.4 (0.3 , 0.7)** | **1651 (1016 , 2509)** | **0.5 (0.3 , 0.7)** | **1.7 (0.5 , 2.9)** |
| **Burundi** | **21 (13 , 32)** | **0.4 (0.3 , 0.7)** | **46 (28 , 70)** | **0.4 (0.3 , 0.7)** | **0.2 (-0.3 , 0.7)** |
| **Comoros** | **2 (1 , 3)** | **0.4 (0.3 , 0.7)** | **3 (2 , 5)** | **0.4 (0.3 , 0.7)** | **-0.1 (-0.3 , 0.1)** |
| **Djibouti** | **2 (1 , 3)** | **0.4 (0.3 , 0.7)** | **5 (3 , 7)** | **0.4 (0.3 , 0.7)** | **0.1 (-0.2 , 0.3)** |
| **Eritrea** | **11 (7 , 17)** | **0.4 (0.3 , 0.7)** | **26 (16 , 40)** | **0.4 (0.3 , 0.7)** | **0.1 (0 , 0.3)** |
| **Ethiopia** | **217 (132 , 332)** | **0.5 (0.3 , 0.7)** | **456 (278 , 694)** | **0.5 (0.3 , 0.7)** | **0.2 (0 , 0.4)** |
| **Kenya** | **110 (67 , 170)** | **0.5 (0.3 , 0.8)** | **245 (150 , 372)** | **0.5 (0.3 , 0.8)** | **-0.3 (-0.5 , -0.1)** |
| **Madagascar** | **46 (28 , 70)** | **0.4 (0.3 , 0.7)** | **104 (64 , 158)** | **0.4 (0.3 , 0.7)** | **-0.3 (-0.5 , -0.1)** |
| **Malawi** | **36 (22 , 56)** | **0.4 (0.3 , 0.7)** | **71 (43 , 108)** | **0.4 (0.3 , 0.7)** | **-0.3 (-0.5 , -0.2)** |
| **Mozambique** | **51 (31 , 77)** | **0.4 (0.3 , 0.7)** | **113 (69 , 172)** | **0.4 (0.3 , 0.7)** | **-0.2 (-0.4 , 0)** |
| **Rwanda** | **27 (17 , 42)** | **0.4 (0.3 , 0.7)** | **50 (31 , 76)** | **0.4 (0.3 , 0.7)** | **-0.2 (-0.4 , -0.1)** |
| **Somalia** | **27 (17 , 41)** | **0.4 (0.3 , 0.7)** | **77 (47 , 117)** | **0.4 (0.3 , 0.7)** | **-0.2 (-0.6 , 0)** |
| **South Sudan** | **22 (14 , 34)** | **0.4 (0.3 , 0.7)** | **36 (22 , 55)** | **0.4 (0.3 , 0.7)** | **-0.1 (-0.7 , 0.3)** |
| **United Republic of Tanzania** | **78 (46 , 117)** | **0.3 (0.2 , 0.5)** | **194 (118 , 296)** | **0.4 (0.2 , 0.6)** | **16.8 (6.1 , 29.9)** |
| **Uganda** | **65 (40 , 99)** | **0.4 (0.3 , 0.7)** | **155 (95 , 238)** | **0.4 (0.3 , 0.7)** | **-0.4 (-0.7 , -0.1)** |
| **Zambia** | **30 (18 , 46)** | **0.4 (0.3 , 0.7)** | **70 (43 , 106)** | **0.4 (0.3 , 0.7)** | **-0.4 (-0.8 , -0.1)** |
| **Central Sub-Saharan Africa** | **350 (216 , 524)** | **0.7 (0.4 , 1.1)** | **839 (522 , 1257)** | **0.7 (0.4 , 1.1)** | **-0.2 (-0.5 , 0)** |
| **Angola** | **65 (40 , 97)** | **0.7 (0.4 , 1.1)** | **190 (118 , 285)** | **0.7 (0.4 , 1.1)** | **-0.7 (-1.1 , -0.3)** |
| **Central African Republic** | **17 (11 , 26)** | **0.7 (0.4 , 1.1)** | **34 (21 , 51)** | **0.7 (0.4 , 1.1)** | **-0.1 (-0.4 , 0.1)** |
| **Congo** | **15 (10 , 23)** | **0.7 (0.4 , 1.1)** | **35 (22 , 52)** | **0.7 (0.4 , 1.1)** | **0.5 (0.2 , 0.8)** |
| **Democratic Republic of the Congo** | **243 (150 , 364)** | **0.7 (0.4 , 1.1)** | **559 (348 , 840)** | **0.7 (0.4 , 1.1)** | **-0.2 (-0.6 , 0.1)** |
| **Equatorial Guinea** | **3 (2 , 4)** | **0.7 (0.4 , 1.1)** | **9 (6 , 13)** | **0.7 (0.4 , 1.1)** | **0.5 (0 , 1.1)** |
| **Gabon** | **6 (4 , 10)** | **0.7 (0.4 , 1.1)** | **12 (7 , 18)** | **0.7 (0.4 , 1.1)** | **0 (-0.3 , 0.3)** |
