# Supplementary figures and images for: Global, regional, and national burden of Guillain–Barré syndrome and its underlying causes from 1990 to 2019
Source: J Neuroinflammation. 2021 Nov 11;18:264. doi: 10.1186/s12974-021-02319-4 (PMC8581128; doi:10.1186/s12974-021-02319-4)

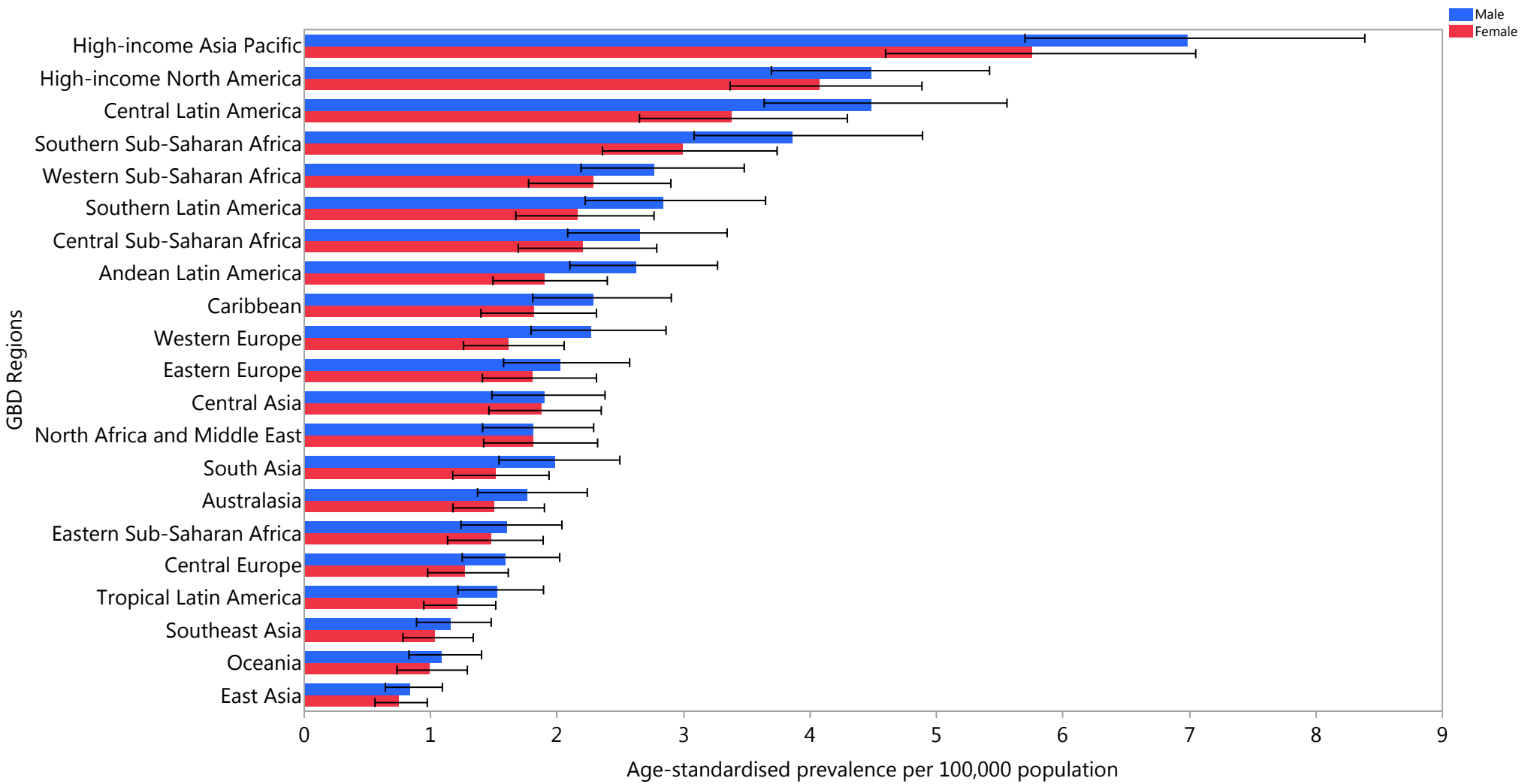

Supplement: Supplementary file 4 — Additional file 4: Figure S1. The age-standardised point prevalence of Guillain–Barre syndrome in 2019 for the 21 Global Burden of Disease regions, by sex. The error bars represent 95% uncertainty intervals for the age-standardised prevalence per 100,000 population. (Generated from data available from http://ghdx.healthdata.org/gbd-results-tool). [file 12974_2021_2319_MOESM4_ESM.pdf]

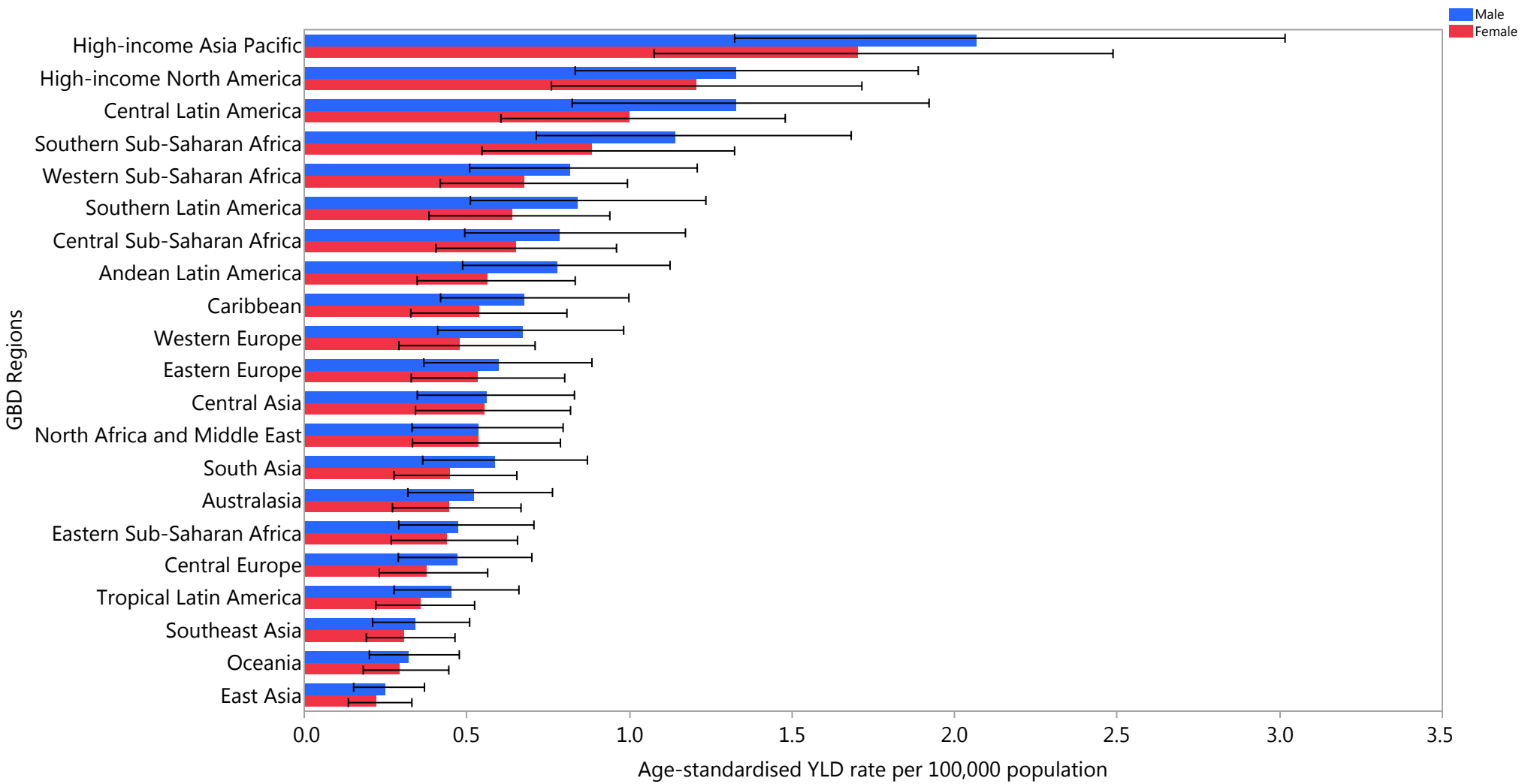

Supplement: Supplementary file 5 — Additional file 5: Figure S2. The age-standardised years lived with disability (YLDs) rates of Guillain–Barre syndrome in 2019 for the 21 Global Burden of Disease regions, by sex. The error bars represent 95% uncertainty intervals for the age-standardised YLD rate per 100,000 population. (Generated from data available from http://ghdx.healthdata.org/gbd-results-tool). [file 12974_2021_2319_MOESM5_ESM.pdf]

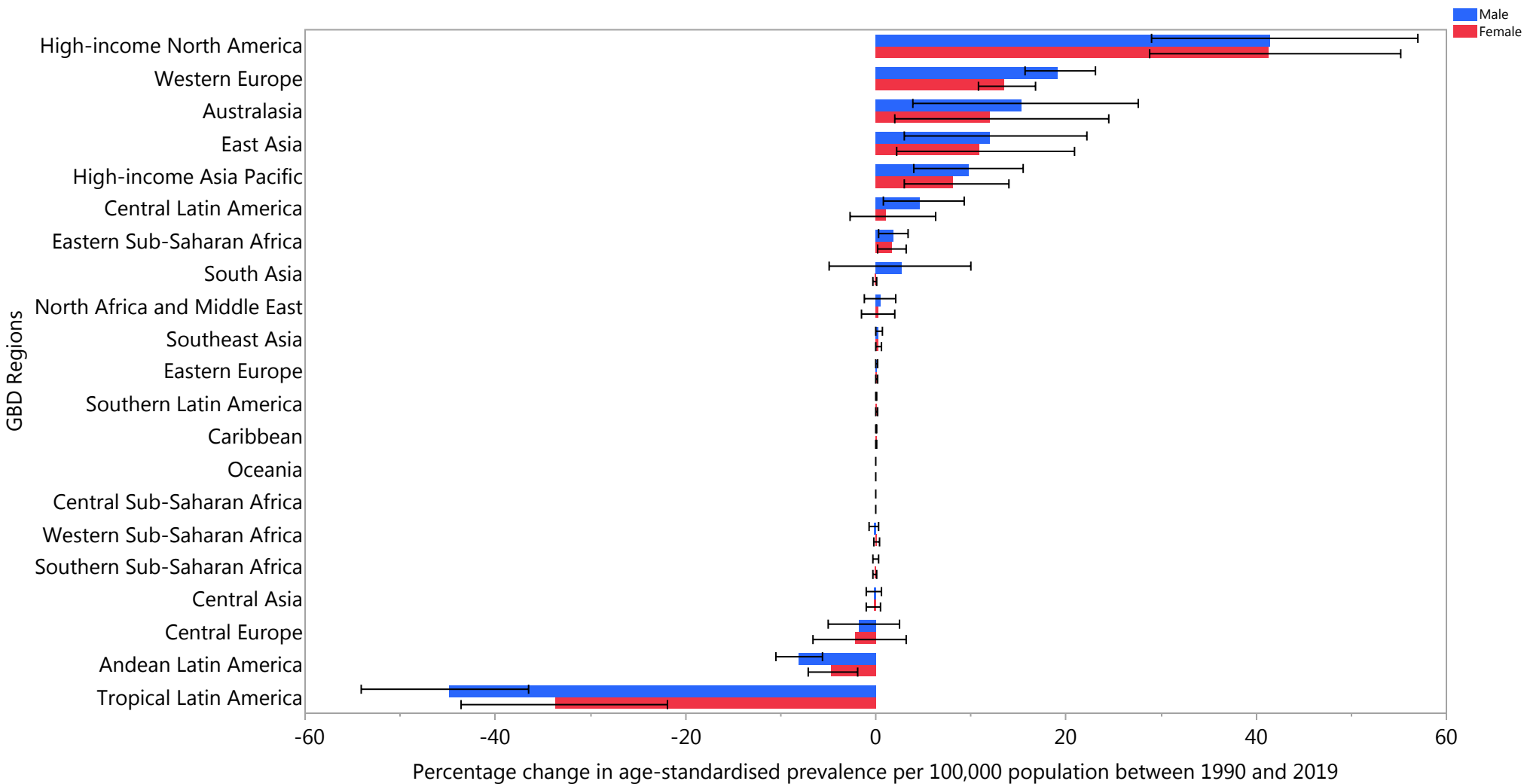

Supplement: Supplementary file 6 — Additional file 6: Figure S3. The percentage change in the age-standardised point prevalence of Guillain–Barre syndrome from 1990 to 2019 for the 21 Global Burden of Disease regions, by sex. The error bars represent 95% uncertainty intervals for the percentage change in age-standardised prevalence per 100,000 population between 1990 and 2019. (Generated from data available from http://ghdx.healthdata.org/gbd-results-tool). [file 12974_2021_2319_MOESM6_ESM.pdf]

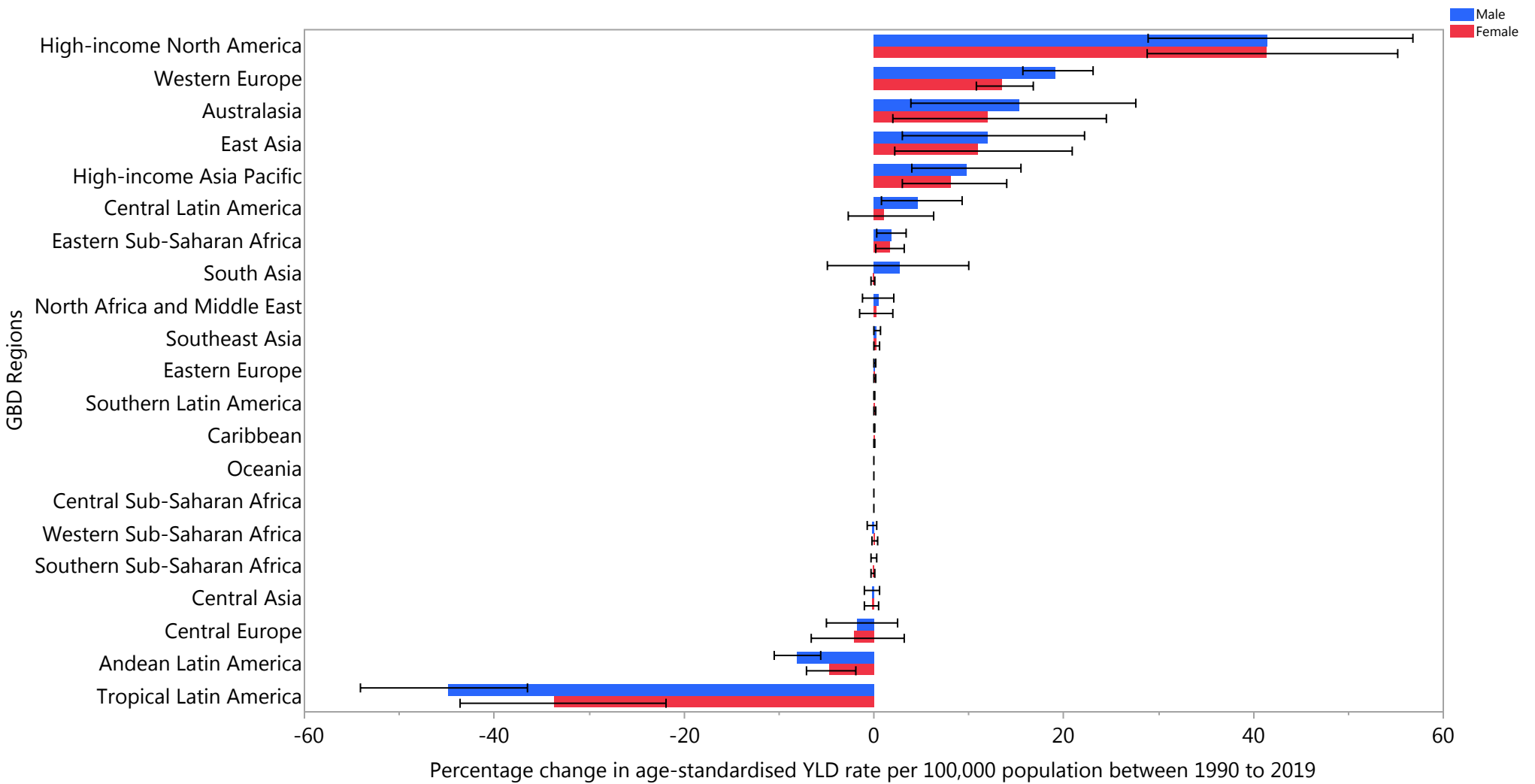

Supplement: Supplementary file 7 — Additional file 7: Figure S4. The percentage change in the age-standardised years lived with disability (YLDs) rates of Guillain–Barre syndrome from 1990 to 2019 for the 21 Global Burden of Disease regions, by sex. The error bars represent 95% uncertainty intervals for the percentage change in age-standardised YLD rates per 100,000 population between 1990 and 2019. (Generated from data available from http://ghdx.healthdata.org/gbd-results-tool). [file 12974_2021_2319_MOESM7_ESM.pdf]

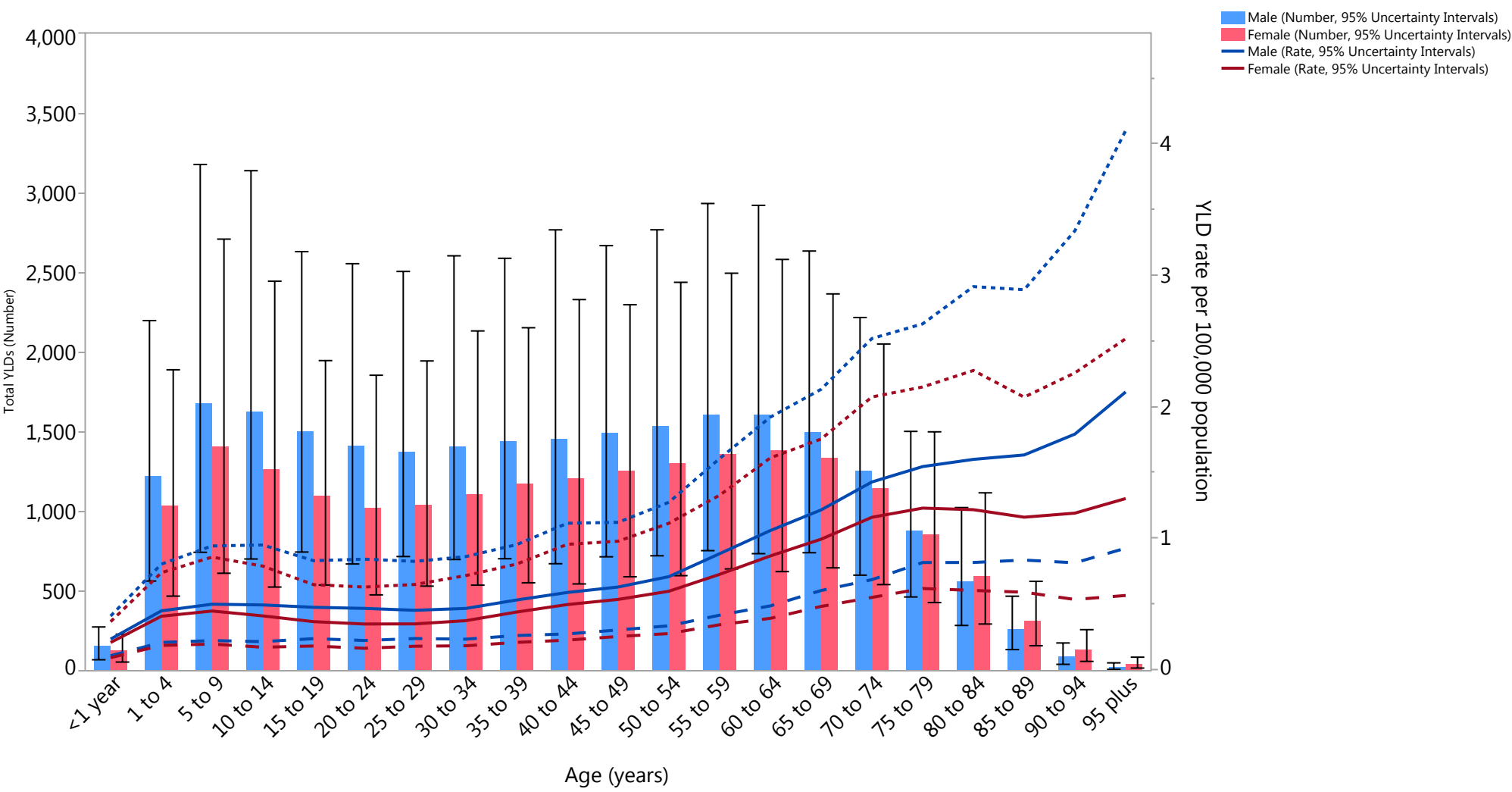

Supplement: Supplementary file 8 — Additional file 8: Figure S5. Global number of years lived with disability (YLDs) cases and years lived with disability (YLDs) of Guillain–Barre syndrome per 100,000 population, by age and sex in 2019. The error bars represent 95% uncertainty intervals for total number of YLDs. Dotted and dashed lines indicate 95% uncertainty intervals for YLD (per 100,000). (Generated from data available from http://ghdx.healthdata.org/gbd-results-tool). [file 12974_2021_2319_MOESM8_ESM.pdf]

Age-standardised YLD rate per 100,000 population

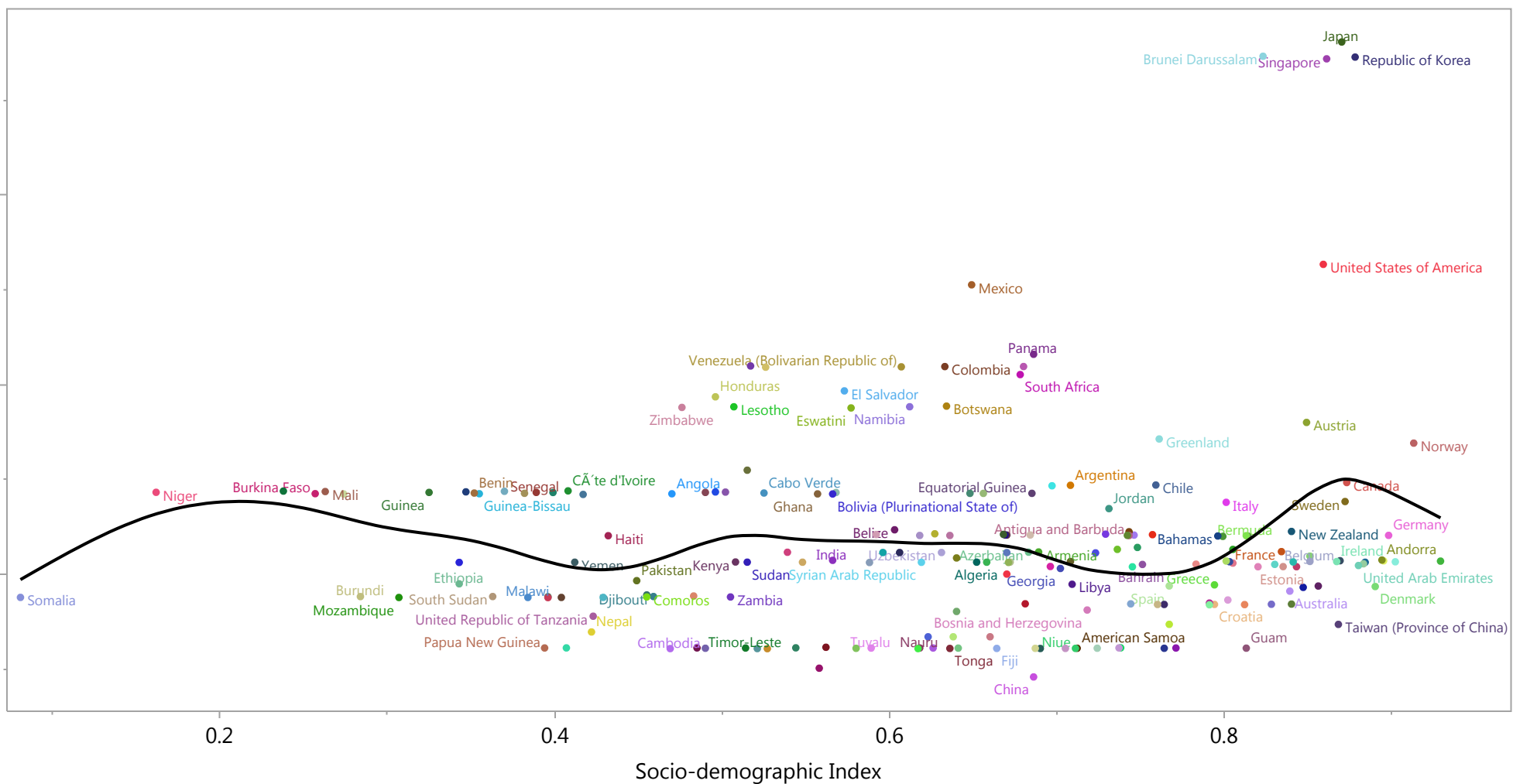

Supplement: Supplementary file 9 — Additional file 9: Figure S6. Age-standardised prevalence rates of Guillain–Barre syndrome for 204 countries and territories, by Socio-demographic Index (SDI), in 2019; Expected values based on the Socio-demographic Index and disease rates in all locations are shown as the black line. Each point shows the observed age-standardised YLD rate for each country in 2019. (Generated from data available from http://ghdx.healthdata.org/gbd-results-tool). [file 12974_2021_2319_MOESM9_ESM.pdf]

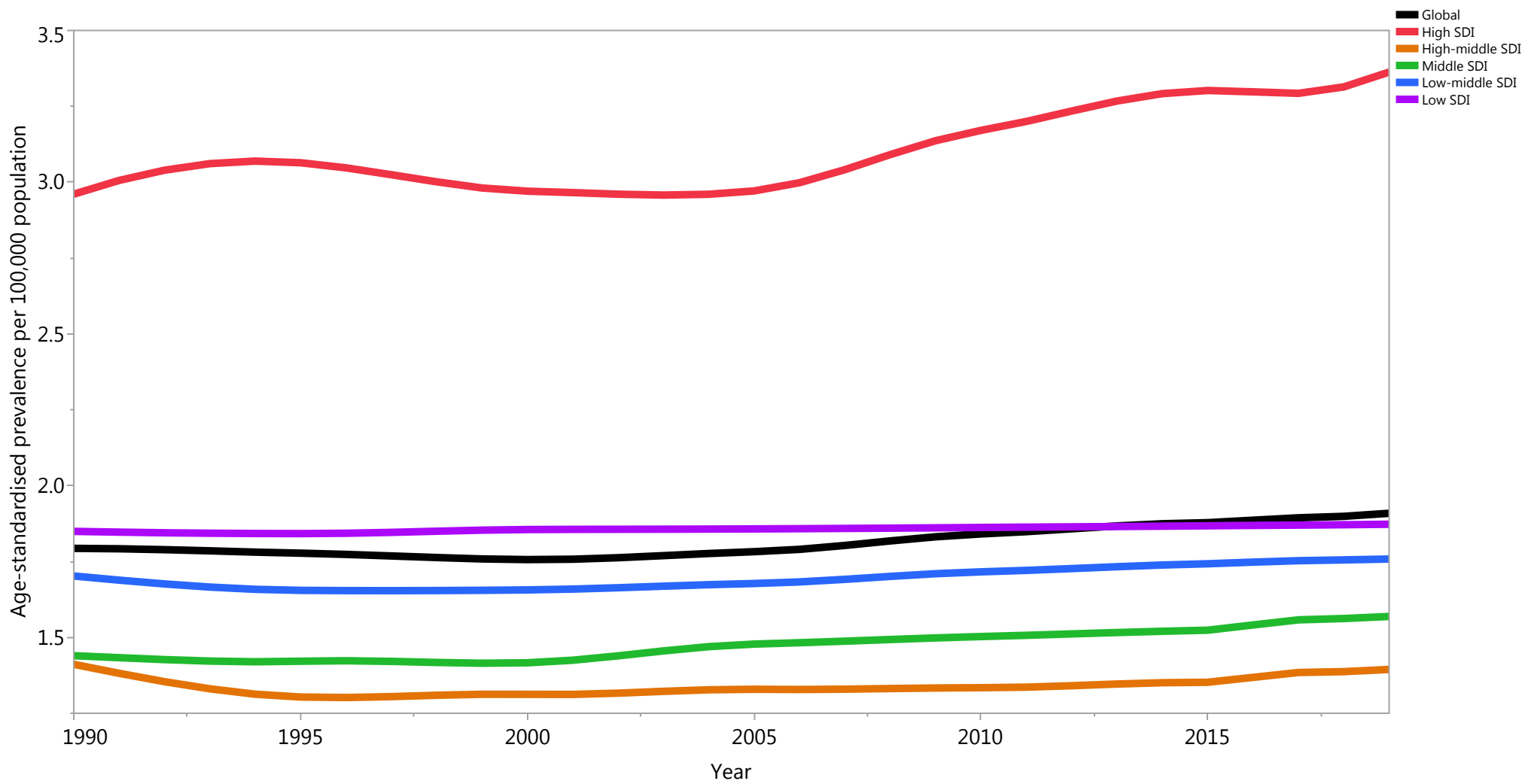

Supplement: Supplementary file 10 — Additional file 10: Figure S7. Age-standardised prevalence rates of Guillain–Barre syndrome, by Socio-demographic Index (SDI) quintiles, from 1990 to 2019; (Generated from data available from http://ghdx.healthdata.org/gbd-results-tool). [file 12974_2021_2319_MOESM10_ESM.pdf]

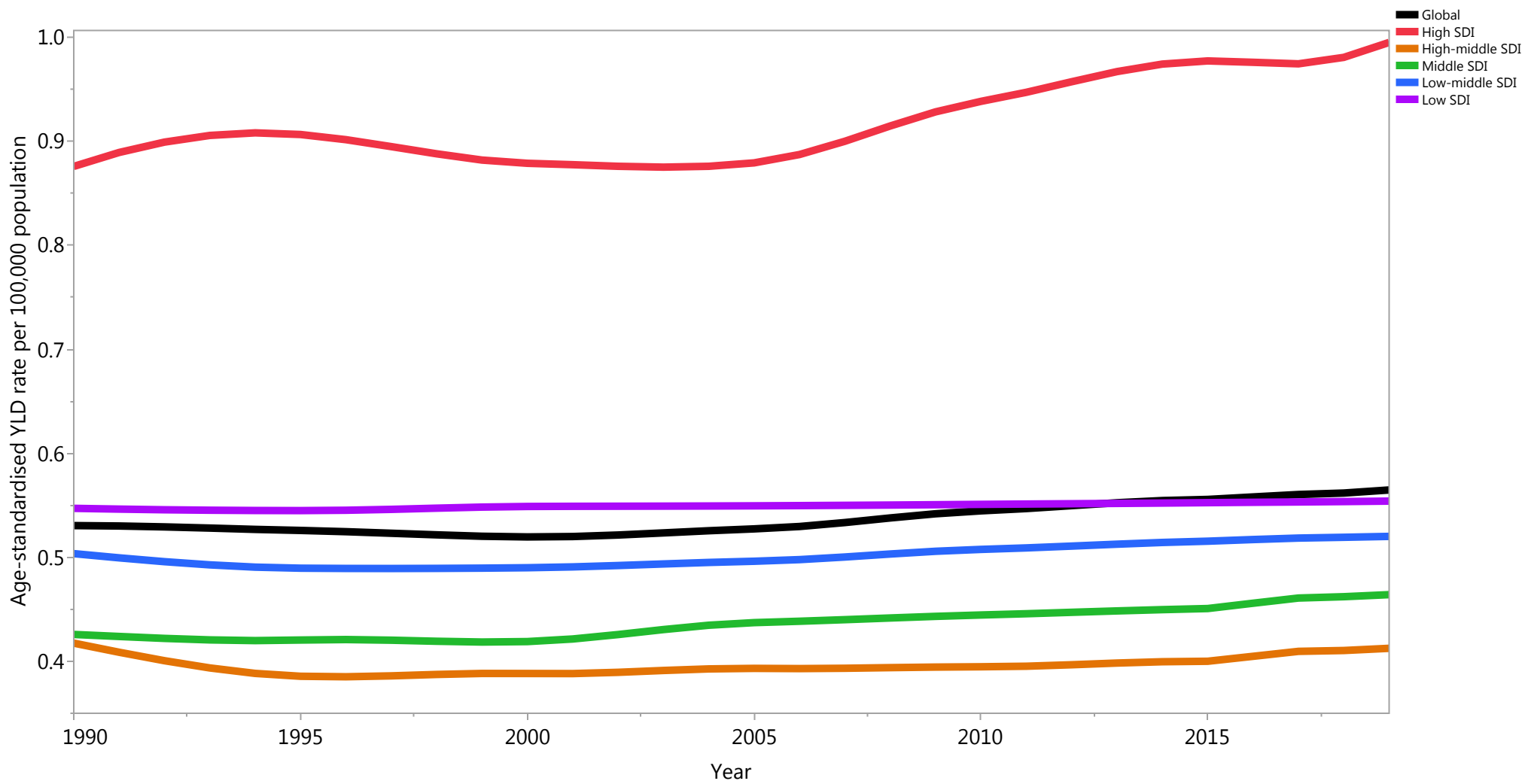

Supplement: Supplementary file 11 — Additional file 11: Figure S8. Age-standardised years lived with disability (YLDs) rates of Guillain–Barre syndrome, by Socio-demographic Index (SDI) quintiles, from 1990 to 2019; (Generated from data available from http://ghdx.healthdata.org/gbd-results-tool). [file 12974_2021_2319_MOESM11_ESM.pdf]

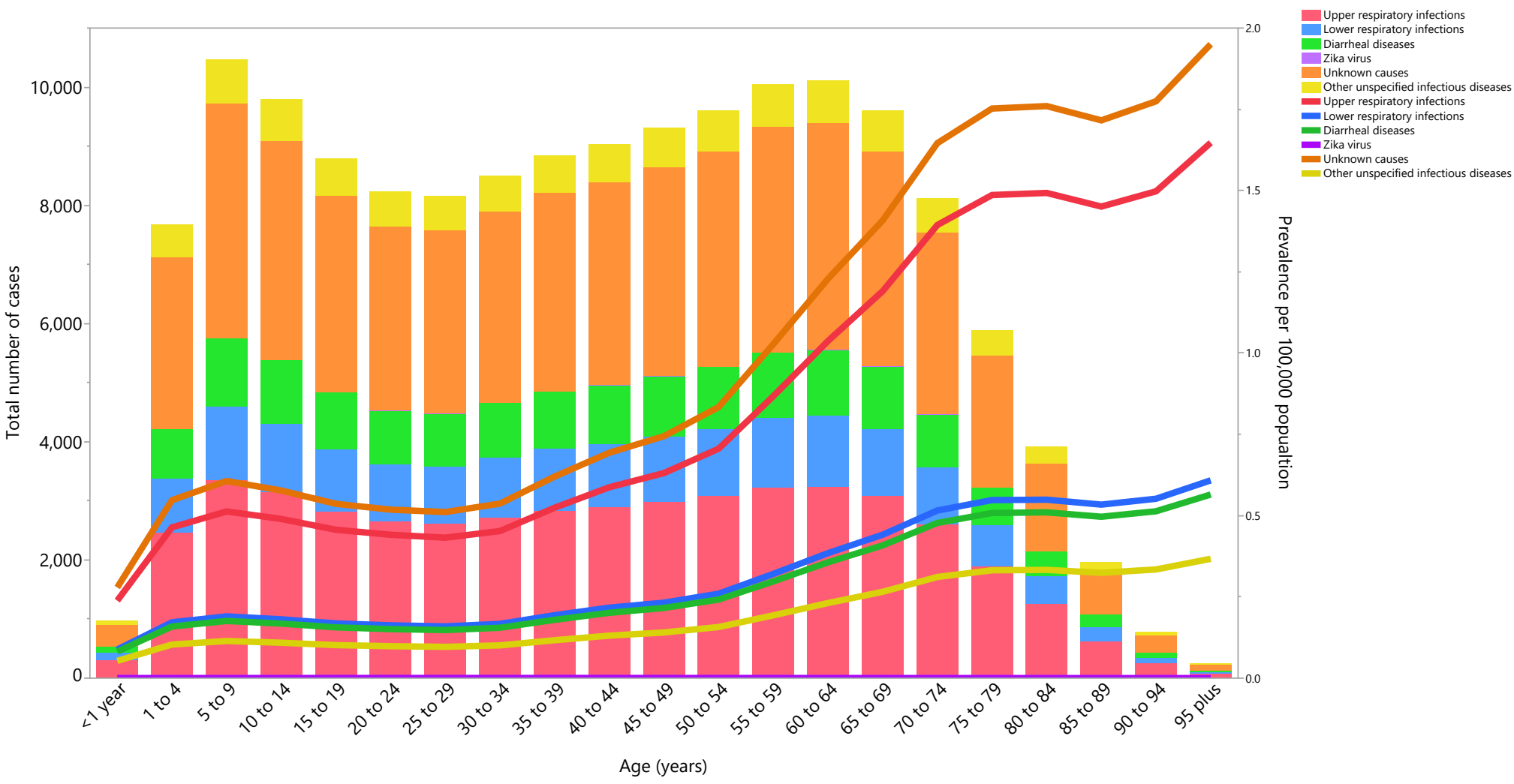

Supplement: Supplementary file 12 — Additional file 12: Figure S9. Global total number of cases and prevalence due to Guillain–Barre syndrome per 100,000 population attributable to each underlying cause, by age in 2019. (Generated from data available from http://ghdx.healthdata.org/gbd-results-tool). [file 12974_2021_2319_MOESM12_ESM.pdf]
